# Clinical trial-identified inflammatory biomarkers in breast and pancreatic cancers

**DOI:** 10.3389/fendo.2023.1106520

**Published:** 2023-04-27

**Authors:** Jing Peng, Supradeep Madduri, Angela D. Clontz, Delisha A. Stewart

**Affiliations:** ^1^ Department of Nutrition, University of North Carolina at Chapel Hill, Chapel Hill, NC, United States; ^2^ Nutrition Research Institute, University of North Carolina at Chapel Hill, Kannapolis, NC, United States; ^3^ Department of Nutrition, Meredith College, Raleigh, NC, United States

**Keywords:** inflammatory molecules, breast cancer, pancreatic cancer, diagnostic biomarkers, treatment biomarkers, prognostic biomarkers

## Abstract

Breast cancer and pancreatic cancer are two common cancer types characterized by high prevalence and high mortality rates, respectively. However, breast cancer has been more well-studied than pancreatic cancer. This narrative review curated inflammation-associated biomarkers from clinical studies that were systematically selected for both breast and pancreatic cancers and discusses some of the common and unique elements between the two endocrine-regulated malignant diseases. Finding common ground between the two cancer types and specifically analyzing breast cancer study results, we hoped to explore potential feasible methods and biomarkers that may be useful also in diagnosing and treating pancreatic cancer. A PubMed MEDLINE search was used to identify articles that were published between 2015-2022 of different kinds of clinical trials that measured immune-modulatory biomarkers and biomarker changes of inflammation defined in diagnosis and treatment of breast cancer and pancreatic cancer patients. A total of 105 papers (pancreatic cancer 23, breast cancer 82) were input into Covidence for the title and abstract screening. The final number of articles included in this review was 73 (pancreatic cancer 19, breast cancer 54). The results showed some of the frequently cited inflammatory biomarkers for breast and pancreatic cancers included IL-6, IL-8, CCL2, CD8+ T cells and VEGF. Regarding unique markers, CA15-3 and TNF-alpha were two of several breast cancer-specific, and CA19 and IL-18 were pancreatic cancer-specific. Moreover, we discussed leptin and MMPs as emerging biomarker targets with potential use for managing pancreatic cancer based on breast cancer studies in the future, based on inflammatory mechanisms. Overall, the similarity in how both types of cancers respond to or result in further disruptive inflammatory signaling, and that point to a list of markers that have been shown useful in diagnosis and/or treatment method response or efficacy in managing breast cancer could potentially provide insights into developing the same or more useful diagnostic and treatment measurement inflammatory biomarkers for pancreatic cancer. More research is needed to investigate the relationship and associated inflammatory markers between the similar immune-associated biological mechanisms that contribute to breast and pancreatic cancer etiology, drive disease progression or that impact treatment response and reflect survival outcomes.

## 1 Introduction

Breast cancer is the world’s most prevalent cancer and in the past five years approximately 2.3 million women have been diagnosed resulting in 685,000 deaths globally through 2020 (1). In the US, the estimated number of new breast cancer cases is 287,850, representing 15% of all new cancer cases (2). Approximately 90% of breast cancer incidence is considered spontaneous or sporadic, whereas the other 5-10% is associated with specific genetic mutations and give rise to hereditary-linked disease (2, 3). The primary genes associated with hereditary breast cancer are BReast CAncer gene 1 (BRCA1) and BRCA2, which produce proteins involved in DNA damage repair. While mutations in these genes are only detected in 2-3% of all breast cancer cases, they increase the risk for developing the disease by 20 to 75% for carriers, between the ages of 40 years up to 70 years old, respectively (4). BRCA1 and BRCA2 were the first two genes identified as having been associated with increased risk for breast cancer, when there is a mutation of either one (5). Thus, beyond genetic predisposition from family history, the high prevalence of disease burden is greatly influenced by other lifestyle-derived risk factors, many of which are modifiable, including nutritional (i.e., alcohol consumption), having obesity, being more sedentary compared to being more active, and others that increase the risk (2). In contrast, pancreatic cancer is not as prevalent as breast cancer but is still the 12^th^ most common cancer worldwide (6). However, it is known as one of the most lethal forms of cancer because most patients usually do not have any symptoms at the early stage, depending on the subtype (7). Thus, unfortunately by the time diagnosis occurs, there are limited treatment options and a poorer prognosis. In 2019, an estimated 89,248 people lived with pancreatic cancer in the United States, and the 5-year survival rate was only 11.5% (8), whereas compared to breast cancer, subtype-dependent survival rates can be up to 91% (2). Like with breast cancer, hereditary and especially having multi-member family history incidence is a prevalent risk factor in pancreatic cancer (6). But still, this too only accounts for ~10% of the total disease burden (9), whereas, smoking is the most strongly linked lifestyle factor known to increase risk in up to 25% of cases, followed by other factors such as having obesity and being tall (6). Also similar to breast cancer, pancreatic cancer has several primary genes related to driving disease, including Kirsten rat sarcoma virus 2(KRAS2), cyclin-dependent kinase inhibitor 2A (p16/CDKN2A), tumor protein 53 (TP53), and mothers against decapentaplegic homolog 4 (SMAD4/DPC4) (10, 11). In addition, recent research has shown that known mutations in the BRCA genes also contribute to increased risk of pancreatic cancer (12, 13).

Although breast cancer and pancreatic cancer are two unique cancer types, they still have some similarities based on endocrine regulation. Namely, and for the focus of this review, hormones play an important role in both cancer types. Estrogen and progesterone are the hormones that primarily influence breast cancer, while Cholecystokinin (CCK, a key hormone that inhibits stomach emptying and stimulates midgut motility in gastric species) is involved in pancreatic cancer development, where researchers have found the CCK-receptor expressed in pancreatic cancer cells (14, 15), in the same way that the estrogen and progesterone receptors are present on the surface of many cell types, including those of the breast (16). Moreover, there is increasing interest in exploring the effect of the sex hormones, estrogen and progesterone, on treating pancreatic cancer, where lessons could be learned from research and clinical practice for breast cancer (15). Although there are several other cancers that fall under endocrine regulation and are driven by the presence or absence of hormones (i.e., cervical/endometrial/uterine cancers, ovarian cancer, and prostate cancer), we focused this comparative review on breast cancer, one of the most widely studied cancers to pancreatic cancer, one with the worst 5-year survival outcomes. In addition, we specialized the content of the comparison on another mechanism, beyond endocrine regulation, greatly influencing the status of both cancer types being inflammation. For breast cancer, the related factors include several chemokines, pro-inflammatory cytokines, adipokines, and hormone receptor growth factors (i.e., insulin-like growth factor) with immuno-modulatory function (17). Common inflammation-associated biomarkers shown to play a role in breast cancer diagnosis and treatment include human epidermal growth factor receptor 2 (HER2), programmed death ligand-1 (PD-L1), cluster of differentiation 4 (CD4), cluster of differentiation 6 (CD6), Interleukin 1 beta (IL-1β), Interleukin 6 (IL-6), Interleukin 8 (IL-8), Insulin-like growth factor 1 (IGF-1), and vascular endothelial growth factor (VEGF), etc. Regarding disease development and progression of pancreatic cancer, important inflammation-related markers of consequence include nuclear factor kappa light enchain enhancer of activated B cells (NF-κB), tumor necrosis factor alpha (TNF-α), Interleukin 1 alpha (IL-1α), Interleukin 4 (IL-4), IL-6, IL-8, IL-1β, and cyclooxygenase-2 (COX-2), etc. (18). As can already be seen in the brief lists above, certain inflammatory biomarkers including several interleukins are relevant to both types of cancer. Both chronic systemic inflammatory changes prior to development of cancer and more associated with lifestyle risk factors, as well as those caused by the tumor microenvironment, directed by these molecules can lead to increased risks and track poorer outcomes for women with breast cancer, and enhance the risk for all patients with pancreatic adenocarcinoma (19, 20). Considering the influence of genetic contributions, although the currently accepted primary genes involved with these two diseases are for the most part different, the mounting evidence of BRCA gene mutations in both cancer types is interesting and will be further discussed in the context of inflammation during this review. Yet the critical question we sought to answer is of the many inflammatory markers associated with either and both diseases, which are the most clinically important?

Due to the similarities between breast and pancreatic cancers, we believed there is great potential to translate the vast amounts of knowledge already learned from breast cancer, some of which has been put into practice, to better inform about how to proceed for pancreatic cancer. Using information about how inflammatory mechanisms drive or impact the unique and/or similar features and phenotypes of these two cancers, coupled with identified inflammation-associated biomarkers, tested out at the level of clinical trials and found to have utility in being able to guide diagnostic and treatment modalities, could hasten improved outcomes for pancreatic cancer patients. To date, no such review of the literature for this specific information has been performed. Thus, the primary purpose of this review is to explore the differences and similarities in inflammation-based biomarkers of breast and pancreatic cancer and discuss the relevance of analyzing and learning from breast cancer studies to potentially improve diagnosis and treatment strategies in pancreatic cancer. The review lists all inflammation biomarkers curated from studies based on a set of clear criteria, separates them into cancer type specific tables, then the unique and shared biomarkers for both cancer types. We discuss how many of them are similar or different in action or clinical implication, as well as discuss markers currently only found to have utility in breast cancer and how they could be relevant for future research activities to determine clinical utility in pancreatic cancer. This comparative review is important because in spite of the many markers that have already been associated with pancreatic cancer (21, 22), there are still many unknowns regarding all the molecules within the inflammatory landscape that could serve as a readout for earlier detection and diagnosis, disease progression and optimal treatment strategies for pancreatic cancer. Using a systematic process, we reviewed clinical trial data from a narrow range of the most recent literature at the time we began, between 2015 through 2022 and listed both breast cancer-specific and pancreatic cancer-specific markers and summarized all relevant markers that were evaluated during that time-range as being considered for use or currently in use for clinical measures of diagnosis, treatment or were linked to and being used to evaluate survival outcomes. Our review with the corresponding tables provides the field with a specific up-to-date guide of how the mechanism of inflammation, as a hallmark of cancer (23) and its mediators have been utilized to improved health outcomes for both breast and pancreatic cancer patients; but further discusses the potential importance of several inflammation biomarkers only identified in breast-cancer, at this time, to serve as a roadmap toward improving diagnosis and treatment in pancreatic cancer.

## 2 Methods

### 2.1 Search strategy and selection criteria

A systematic search strategy was developed and conducted through the Health Sciences Library (HSL) at the University of North Carolina at Chapel Hill. A list of relevant key words was curated for both pancreatic cancer and breast cancer using a PubMed mesh term approach (https://www.ncbi.nlm.nih.gov/mesh/), including the following: “Cancer antigen (CA)-19-9 Antigen, pancreatitis-Associated Proteins, Chemokines, Interleukins, Lymphokines, Monokines, Tumor Necrosis Factors, and Pancreatic Neoplasms” and “CA-125 Antigen, Mucin-1, Mammaglobin A, Chemokines, Interleukins, Lymphokines, Monokines, Tumor Necrosis Factors, and Breast Neoplasms,” respectively. Male Breast cancer was also searched separately by adding the key word “Breast Neoplasms, Male”. The complete list of search terms was built by Search Builder provided through HSL (**Table 1**). All initial studies retrieved from the three separate searches were uploaded to Covidence systematic review software for title and abstract screening. Duplicate citations were eliminated during this stage of the screening process. Potentially eligible studies were retrieved in full and reviewed independently by the first author. Two additional rounds of full text data abstraction and quality control verification were subsequently performed by the other three authors.

**Table 1:** Complete list of search terms for Reviewed Cancer Types from PubMed mesh term search.

Cancer Type	Searching Term
**Pancreatic Cancer**	(Pancreatic Neoplasms[mesh] OR Pancreatic Neoplasms[tiab] OR Neoplasm, Pancreatic[tiab] OR Pancreatic Neoplasm[tiab] OR Neoplasm, Pancreas[tiab] OR Pancreas Neoplasm[tiab] OR Cancer of Pancreas[tiab] OR Pancreas Cancers[tiab] OR Pancreas Cancer[tiab] OR Cancer, Pancreas[tiab] OR Cancers, Pancreas[tiab] OR Pancreatic Cancer[tiab] OR Cancer, Pancreatic[tiab] OR Pancreatic Cancers[tiab] OR Cancer of the Pancreas[tiab]) AND (CA-19-9 Antigen[mesh] OR CA-19-9 Antigen[tiab] OR Antigen, CA-19-9[tiab] OR CA 19 9 Antigen[tiab] OR Sialyl Le(a)[tiab] OR CA 19-9 Antigen[tiab] OR alpha-Neu5Ac-(2-3)-beta-D-Gal-(1-3)-(alpha-L-Fuc-(1-4))-beta-D-GlcNAc[tiab] OR NAG-1,3-F-1,4-GN[tiab] OR Neu5Ac-2-3-Gal-1-3-(Fuc-1-4)-GlcNAc[tiab] OR Sialyl Lewis A[tiab] OR A, Sialyl Lewis[tiab] OR Sialyl Lewis(a) Tetrasaccharide[tiab] OR Sialyl Lewis A Antigen[tiab] OR Gastrointestinal Cancer Antigen[tiab] OR Cancer Antigen, Gastrointestinal[tiab] OR Pancreatitis-Associated Proteins[mesh] OR Pancreatitis-Associated Proteins[tiab] OR Regenerating Islet-Derived Protein[tiab] OR Islet-Derived Protein, Regenerating[tiab] OR Protein, Regenerating Islet-Derived[tiab] OR Regenerating Islet-Derived Proteins[tiab] OR Pancreatitis-Associated Protein-1[tiab] OR Pancreatitis Associated Protein[tiab] OR Regenerating Islet-Derived Protein 3[tiab] OR Chemokines[mesh] OR Chemokines[tiab] OR Chemotactic Cytokine[tiab] OR Cytokine, Chemotactic[tiab] OR Intercrines[tiab] OR Chemotactic Cytokines[tiab] OR Cytokines, Chemotactic[tiab] OR Intercrine[tiab] OR Chemokine[tiab] OR Interleukins[mesh] OR Interleukins[tiab] OR Lymphokines[mesh] OR Lymphokines[tiab] OR Lymphocyte Mediators[tiab] OR Mediators, Lymphocyte[tiab] OR Monokines[mesh] OR Monokines[tiab] OR Tumor Necrosis Factors[mesh] OR Tumor Necrosis Factors[tiab] OR Necrosis facctors, Tumor[tiab] OR TNF Receptor Ligands[tiab] OR Receptor Ligands, TNF[tiab] OR Tumor Necrosis Factor Superfamily Ligands[tiab])
**Male Breast Cancer**	(Breast Neoplasms, Male[mesh] OR Breast Neoplasms, Male[tiab] OR Male Breast Neoplasm[tiab] OR Neoplasm, Male Breast[tiab] OR Tumors, Breast, Male[tiab] OR Neoplasms, Breast, Male[tiab] OR Neoplasms, Male Breast[tiab] OR Breast Tumor, Male[tiab] OR Breast Tumors, Male[tiab] OR Male Breast Tumor[tiab] OR Male Breast Cancer[tiab] OR Carcinoma, Male Breast[tiab] OR Male Breast Carcinoma[tiab]) AND (Biomarker, Tumor [mesh] OR Biomarker, Tumor [tiab])
**Female Breast Cancer**	(Breast Neoplasms[mesh] OR Breast Neoplasms[tiab] OR Breast Neoplasm[tiab] OR Neoplasm, Breast[tiab] OR Breast Tumors[tiab] OR Breast tumor[tiab] OR Tumor, Breast[tiab] OR Mammary Cancer[tiab] OR Cancers, Mammary[tiab] OR Malignany Neoplasm of Breast[tiab] OR Breast Malignant Neoplasm[tiab] OR Malignant Tumor of Breast[tiab] OR Breast Malignant Tumor[tiab] OR Breast Malignant Tumors[tiab] OR Cancer of Breast[tiab] OR Mammary Carcinoma, Human[tiab] OR Carcinoma, Human Mammary[tiab] OR Human Mammary Carcinomas[tiab] OR Neoplasm, Human Mammary[tiab] OR Human Mammary Neoplasm[tiab] OR Breast Carcinoma[tiab] OR Mammary Neoplasm, Human[tiab] OR Breast Carcinoas[tiab])AND (CA-125 Antigen[mesh] OR CA-125 Antigen[tiab] OR Antigen CA-125[tiab] OR Mucin-16[tiab] OR Mucin 16[tiab] OR CA 125 Antigen[tiab] OR Mucin-1[mesh] OR Mucin-1[tiab] OR CA 15.3 Antigen[tiab] OR Antigen CA-15-3[tiab] OR Antigen CA 15 3[tiab] OR CA-15-3 Antigen[tiab] OR Episialin[tiab] OR CD227 Antigen[tiab] OR Polymorphic Epithelial Mucin[tiab] OR Epithelial Membrane Antigen[tiab] OR Muc1 Mucin[tiab] OR Mucin, Muc 1[tiab] OR Antigen, CD 227[tiab] OR CD227 Antigens[tiab] OR CA 15 3 Antigen[tiab] OR Epithelial Mucin, Polymorphic[tiab] OR Mammaglobin A[mesh] OR Mammaglobin A[tiab] OR Secretoglobin Family 2A Member 2[tiab] OR Mammaglobin 1[tiab] OR Mammaglobin-A[tiab] OR Chemokines[mesh] OR Chemokines[tiab] OR Chemotactic Cytokine[tiab] OR Cytokine, Chemotactic[tiab] OR Intercrines[tiab] OR Chemotactic Cytokines[tiab] OR Cytokines, Chemotactic[tiab] OR Intercrine[tiab] OR Chemokine[tiab] OR Interleukins[mesh] OR Interleukins[tiab] OR Lymphokines[mesh] OR Lymphokines[tiab] OR Lymphocyte Mediators[tiab] OR Mediators, Lymphocyte[tiab] OR Monokines[mesh] OR Monokines[tiab] OR Tumor Necrosis Factors[mesh] OR Tumor Necrosis Factors[tiab] OR Necrosis facctors, Tumor[tiab] OR TNF Receptor Ligands[tiab] OR Receptor Ligands, TNF[tiab] OR Tumor Necrosis Factor Superfamily Ligands[tiab]) NOT (Breast Neoplasms, Male[mesh] OR Breast Neoplasms, Male[tiab] OR Male Breast Neoplasm[tiab] OR Neoplasm, Male Breast[tiab] OR Tumors, Breast, Male[tiab] OR Neoplasms, Breast, Male[tiab] OR Neoplasms, Male Breast[tiab] OR Breast Tumor, Male[tiab] OR Breast Tumors, Male[tiab] OR Male Breast Tumor[tiab] OR Male Breast Cancer[tiab] OR Carcinoma, Male Breast[tiab] OR Male Breast Carcinoma[tiab])

[tiab]- restrict the query to search in the title or abstract of the articles; [mesh]- Medical Subject Headings.

Inclusion criteria for scholarly articles included in this review were ^1^being published between 2015-2022, ^2^randomized controlled trial or clinical trial focusing on identifying cancer-related biomarkers or ^3^biomarkers changed in diagnosis and treatment of ^3a^pancreatic cancer, ^3b^male breast cancer or ^3c^female breast cancer, and finally ^4^human study. Initial exclusion criteria included ^1^being published before 2015, ^2^study designs other than randomized controlled trials or clinicals trials, ^3^animal study, and ^4^lack of information for cancer-related inflammation/immune biomarkers. Additionally, the language was limited to English only publications.

Data collection/abstraction was done by all research authors at different levels. Primary author spear-headed the extracted research information and experimental data from all included references and organized them in Excel. The total number of articles was then divided into three groups and the other authors took responsibility for checking the accuracy and completeness of each assigned reference and the information presented in the tables and text of the review.

## 3 Results

### 3.1 Summary of the literature search

**Figure 1:**
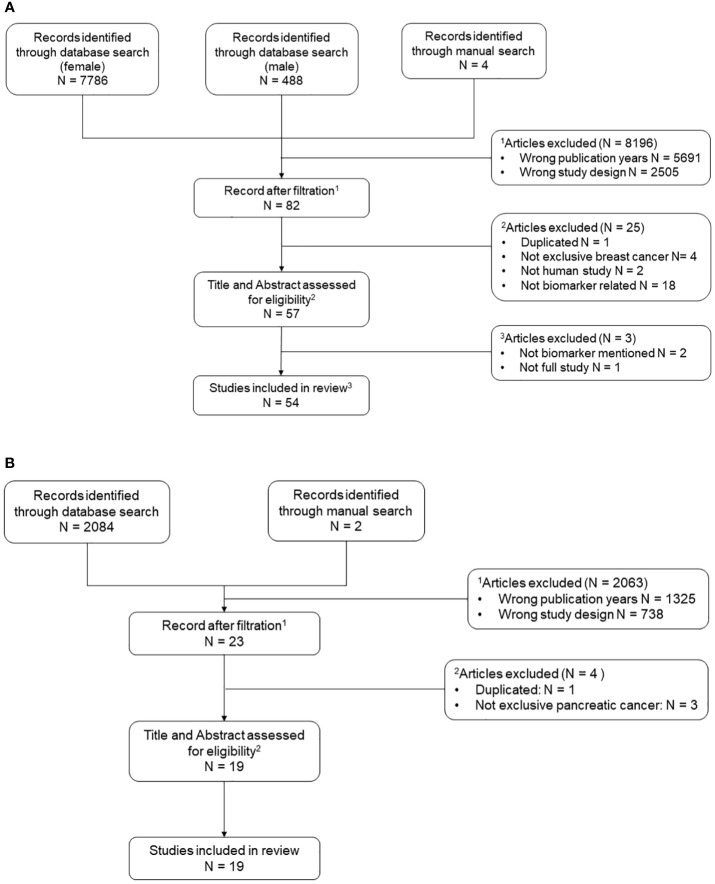
Flow chart of systematic literature search process. N is abbreviation for number. A list of relevant key words was curated for both pancreatic cancer and breast cancer using a PubMed mesh term approach. All initial studies retrieved from the three searches (female breast cancer, male breast cancer, and pancreatic cancer) were uploaded to Covidence for title and abstract screening. Two additional rounds of full text data abstraction and quality control were performed. **(A)** Includes reference selection process for female and male breast cancer. **(B)** Includes reference selection process for pancreatic cancer.

The flow diagram summarizing the systematic process of literature search and selection is shown in **Figure 1**. The National Library of Medicine database PubMed (https://pubmed.ncbi.nlm.nih.gov/) showed 2084 results for pancreatic cancer, 7786 for female breast cancer, and 488 for male breast cancer. In addition, 6 articles were added manually from prior knowledge of the corresponding author based on research expertise. In total, 9876 references were retrieved from the search process. After filtration by publication year and study design, a total of 105 papers (pancreatic cancer 23, breast cancer 82) were input into Covidence for title and abstract screening. After removing duplicates and reviewing each title and abstracts individually, a total of 76 articles remained for full-text review. The final number of articles included in this review is 73 (pancreatic cancer 19, breast cancer 54) due to exclusion of 3 breast cancer papers after the full-text review and data screening processes. Only the inflammation-associated biomarkers determined to show a significant contribution to the study aims or moved forward as a result of the study into clinical practice or moved forward to a subsequent level of test evaluation for clinical utility across all the included studies, are shown in **Table 2**. A more comprehensive summary of the parameters defining all studies reviewed can be found in corresponding **Supplementary Table 1**. The following results sections will only discuss in detail a selection of the most prevalent markers identified in both breast and pancreatic cancers listed in **Table 2**.

**Table 2:** Summary of all studies included in review with details for detected biomarkers.

Name of Study	Study Type	Biomarkers	Ref
Prognostic and predictive value of immunological parameters for chemoradioimmunotherapy in patients with pancreatic adenocarcinoma	clinical trial	Diagnostic: CD4, NK, Neutrophils, Eff CD8, IL-10, MUC CRIt (Chemoradioimmunotherapy) only: CD21+ monocytes, CCR7+, CD21+ lymphocytes, Naïve CD8, eff-mem CD8, CD152+CD4, IL-2, neutrophils, MUC, CD3, CA19 (GrB), CRt (Chemotherapy) only: cytotoxicity, MUC, CA19 (GrB), Eff CD8	(24)
BL-8040, a CXCR4 antagonist, in combination with pembrolizumab and chemotherapy for pancreatic cancer: the COMBAT trial	phase IIa, open-label, two -cohort study	CD4+ CD69+ T cells, CXCR4-expressing CD4+ and CD8+ T cells; Treg cells (CD4+ CD25+ FoxP3+)	(25)
The tumor-targeting immunocytokine F16-IL2 in combination with doxorubicin: dose escalation in patients with advanced solid tumors and expansion into patients with metastatic breast cancer	open-label, non-randomized, phase Ib/II study	Immunogenic response not showed	(26)
Improved Natural Killer cell activity and retained anti-tumor CD8(+) T cell responses contribute to the induction of a pathological complete response in HER2-positive breast cancer patients undergoing neoadjuvant chemotherapy	phase II mono-institutional trial	NK cells, regulatory T cells; T helper 17 cells; CD8+ T cell, CD4+ T cell	(27)
High-circulating Tie2 Is Associated With Pathologic Complete Response to Chemotherapy and Antiangiogenic Therapy in Breast Cancer	prospective phase II study	Tie-2, bFGF	(28)
Mindfulness and its efficacy for psychological and biological responses in women with breast cancer	randomized controlled trial	NK cells (CD3-16+56+); CD3+ & CD3+8+ T-lymphocytes; CD19+ B-lymphocytes, CD19B%	(29)
Binding of circulating anti-MUC1 antibody and serum MUC1 antigen in stage IV breast cancer	clinical trial	IgG, CA 15-3	(30)
Receptor activator of nuclear factor kappa B (RANK) expression in primary breast cancer correlates with recurrence-free survival and development of bone metastases in I-SPY1 (CALGB 150007/150012; ACRIN 6657)	multicenter study	Receptor Activator of Nuclear Factor Kappa B (RANK)	(31)
Dynamic changes of Receptor activator of nuclear factor-κB expression in Circulating Tumor Cells during Denosumab predict treatment effectiveness in Metastatic Breast Cancer	pilot study	RANK in CTCs	(32)
Effects of Molecular Iodine/Chemotherapy in the Immune Component of Breast Cancer Tumoral Microenvironment	clinical trial	TH1, TH17, M0 macrophages, B lymphocytes	(33)
Efficacy and Determinants of Response to HER Kinase Inhibition in HER2-Mutant Metastatic Breast Cancer	clinical trial	HER2, HER3	(34)
Intratumoral Hydrogen Peroxide With Radiation Therapy in Locally Advanced Breast Cancer: Results From a Phase 1 Clinical Trial	clinical trial	IL-1b, IL-4, MTP-1a	(35)
Phase I pilot study of Wilms tumor gene 1 peptide-pulsed dendritic cell vaccination combined with gemcitabine in pancreatic cancer	phase I pilot study	tetramer-positive WT 1-specific T cell; NLR, CRP, IL-8	(36)
Prognostic significance of plasma interleukin-6/-8 in pancreatic cancer patients receiving chemoimmunotherapy	clinical trial	IL-6, IL-8	(37)
Phase II trial of salvage therapy with trabectedin in metastatic pancreatic adenocarcinoma	single-center, prospective, single-arm phase II study	CCL27, CXCL9, CXCL 10, IL-6, and TRAIL; PTX3 and MIF; IL-6, IL-8, CXCL1, IL-Ra, CXCL12, IFNa2, PTX3, HGF, and SCGF	(38)
Systemic immune activity predicts overall survival in treatment naive patients with metastatic pancreatic cancer	phase II clinical trial	higher IL-6, IL-10, CTLA-4 on CD8+ T cell; higher MCP-1,CD45RO+, TIM3 on CD4+ cell	(39)
Immunobiological effects of gemcitabine and capecitabine combination chemotherapy in advanced pancreatic ductal adenocarcinoma	clinical trial	CRP, IL-6, GM-CSF	(40)
Randomized Phase 2 Trial of the Oncolytic Virus Pelareorep (Reolysin) in Upfront Treatment of Metastatic Pancreatic Adenocarcinoma	randomized phase 2 trial	IL-6, IL-8, VEGF, regulatory T cell, CTLA4 on both CD+ and CD8+	(41)
Phase 1b study targeting tumour associated macrophages with CCR2 inhibition plus FOLFIRINOX in locally advanced and borderline resectable pancreatic cancer	single-center, open label, phase Ib clinical trial	inflammatory monocyte (CCR2+); IL-12, TNFa, CD8+ TIL, Helper CD4+; IL-4, IL-10, IL-13, TGF-b, FoxP3 regulatory T-cell	(42)
Pharmacogenomic analyses of sunitinib in patients with pancreatic neuroendocrine tumors	phase IV trial	IL1b	(8)
Phase I clinical trial repurposing all-trans retinoic acid as a stromal targeting agent for pancreatic cancer	phase 1b randomized control trial	FABP5, CRABP2, PTX3	(43)
Plasma IL8 Is a Biomarker for TAK1 Activation and Predicts Resistance to Nanoliposomal Irinotecan in Patients with Gemcitabine-Refractory Pancreatic Cancer	prospective trial study with Expanded Access Program from initial randomized phase III trial	IL8	(44)
Phase 1b study of a small molecule antagonist of human chemokine (C-C motif) receptor 2 (PF-04136309) in combination with nab-paclitaxel/gemcitabine in first-line treatment of metastatic pancreatic ductal adenocarcinoma	phase 1b study	CCL2, CD14 + CCR2+ inflammatory monocytes (IM)	(45)
A phase 1b trial of concurrent immunotherapy and irreversible electroporation in the treatment of locally advanced pancreatic adenocarcinoma	phase 1b clinical trial	T-effector memory cell and PD-L1	(46)
Randomized Phase III Study of FOLFOX Alone or With Pegilodecakin as Second-Line Therapy in Patients With Metastatic Pancreatic Cancer That Progressed After Gemcitabine (SEQUOIA)	Randomized phase III study	IL-18, interferon-y, granzyme B; TGF-beta	(47)
Immunologic and tumor responses of pegilodecakin with 5-FU/LV and oxaliplatin (FOLFOX) in pancreatic ductal adenocarcinoma (PDAC)	multi-institutional, open-label, multiple-cohort, dose -escalation, phase 1b study.	CA19-9	(48)
Circulating biomarkers and outcomes from a randomized phase 2 trial of gemcitabine versus capecitabine-based chemoradiotherapy for pancreatic cancer	randomized phase 2 trial	CCL 5	(49)
Effects of Fresh Yellow Onion Consumption on CEA, CA125 and Hepatic Enzymes in Breast Cancer Patients: A Double-Blind Randomized Controlled Clinical Trial	double-blind randomized controlled clinical trial	ALT, AST; CA125, carcinoembryonic antigen (CEA); ALP	(50)
Targeted T-cell Therapy in Stage IV Breast Cancer: A Phase I Clinical Trial	Phase I clinical trial	Th1, IL-12	(51)
Inflammation and psychosocial factors mediate exercise effects on sleep quality in breast cancer survivors: pilot randomized controlled trial	Pilot randomized controlled trial	Il-6, IL-8, IL-10, TNF-alpha	(52)
ELYPSE-7: a randomized placebo-controlled phase IIa trial with CYT107 exploring the restoration of CD4+ lymphocyte count in lymphopenic metastatic breast cancer patients	randomized placebo-controlled phase I trial	CD4+, CD8+	(53)
A Comparison of Fentanyl and Flurbiprofen Axetil on Serum VEGF-C, TNF-α, and IL-1ß Concentrations in Women Undergoing Surgery for Breast Cancer	clinical trial	VEGF-C, TNF-alpha, IL-1beta	(54)
Effect of Propofol and Desflurane on Immune Cell Populations in Breast Cancer Patients: A Randomized Trial	randomized trial	IL-2/IL-4, CD4+/CD8+; NK cell, leukocytes	(55)
Postsurgical Depressive Symptoms and Proinflammatory Cytokine Elevations in Women Undergoing Primary Treatment for Breast Cancer	clinical trial	IL-1beta, TNF-alpha	(56)
Association of CA27.29 and Circulating Tumor Cells Before and at Different Times After Adjuvant Chemotherapy in Patients with Early-stage Breast Cancer - The SUCCESS Trial	clinical trial	CA27.29 (mucin-1)	(57)
Resistance Exercise and Inflammation in Breast Cancer Patients Undergoing Adjuvant Radiation Therapy: Mediation Analysis From a Randomized, Controlled Intervention Trial	randomized controlled intervention trial	IL-6, Il-6/IL-1a	(58)
Angiogenic cytokines and their influence on circulating tumour cells in sera of patients with the primary diagnosis of breast cancer before treatment	prospective, randomized adjuvant study	sFlt1, PIGF, VEGF, VEGF-C, VEGF-D	(59)
Relationship of inflammatory profile of elderly patients serum and senescence-associated secretory phenotype with human breast cancer cells proliferation: Role of IL6/IL8 ratio	clinical trial	IL-6, IL-8, IL-10	(60)
Testing breast cancer serum biomarkers for early detection and prognosis in pre-diagnosis samples	case-control study	CA15-3; PAI-1, HSP90A	(61)
Tumor cryoablation in combination with natural killer cells therapy and Herceptin in patients with HER2-overexpressing recurrent breast cancer	clinical trial	circulating tumor cells (CTCs), carcino-embryonic antigen (CEA), CA15-3	(62)
An anti-inflammatory dietary intervention to reduce breast cancer recurrence risk: Study design and baseline data	one-year, culinary-based, pilot intervention	CRP, IL-6, IL-8, TNF-alpha; IL-10	(63)
Association between changes in fat distribution and biomarkers for breast cancer	three-armed RCT	High sensitivity CRP (hsCRP), leptin	(64)
Criteria derived from serum markers can precisely evaluate axillary status in breast cancer patients	clinical trial	Matrix metalloproteinase-1, hepatocyte growth factor, chemokine ligand 5	(65)
The effect analysis of CYP2D6 gene polymorphism in the toremifene and tamoxifen treatment in patient with breast cancer	clinical trial	CA 125, CA 153, VEGF, IGF-1	(66)
Increased interleukin-35 expression in tumor-infiltrating lymphocytes correlates with poor prognosis in patients with breast cancer	clinical trial	IL-35	(67)
Adipose tissue inflammation in breast cancer survivors: effects of a 16-week combined aerobic and resistance exercise training intervention	pilot randomized study	ATM M1, IL-6, TNF-alpha; ATM M2, adiponectin	(68)
Benefits in radical mastectomy protocol: a randomized trial evaluating the use of regional anesthesia	single-center, prospective, randomized clinical trial	IL-6, IL-10, IL-1 beta	(69)
Vitamin D Levels, Vitamin D Receptor Polymorphisms, and Inflammatory Cytokines in Aromatase Inhibitor-Induced Arthralgias: An Analysis of CCTG MA.27	phase III adjuvant trial	examined 9 baseline inflammatory cytokines: IL-1β, IL-6, TNF-α, IFN-γ, IL-10, IL-12p70, IL-17, IL-23, and CCL-20	(70)
Prognostic impact of CD4-positive T cell subsets in early breast cancer: a study based on the FinHer trial patient population	randomized controlled trial	CXCL13; FOXP3, CD4	(71)
IL1 Receptor Antagonist Controls Transcriptional Signature of Inflammation in Patients with Metastatic Breast Cancer	clinical trial	IL-1beta	(72)
Reducing postsurgical exudate in breast cancer patients by using San Huang decoction to ameliorate inflammatory status: a prospective clinical trial	prospective clinical trial	TNF-alpha, IL-6, IL-8, IL-2R	(73)
Atezolizumab Plus nab-Paclitaxel in the Treatment of Metastatic Triple-Negative Breast Cancer With 2-Year Survival Follow-up: A Phase 1b Clinical Trial	Phase 1b clinical trial	programmed heath-ligand 1, tumor-infiltrating lymphocytes, CD8; CD8+ T cell, CXCL10	(74)
The Anti-tumoral Effect of β-D-Mannuronic Acid (M2000) as a Novel NSAID on Treg Cells Frequency and MMP-2, MMP-9, CCL22 and TGFβ1 Gene Expression in Pre-surgical Breast Cancer Patients	phase II, randomized, controlled trial	MMP-2, MMP-9, CCL22, TGF-beta 1	(75)
Sapylin (OK-432) alters inflammation and angiogenesis *in vivo* and *vitro*	prospective, consecutive cohort study	IL-1a, IL-6, TGF-beta 1, VEGF/HUVEC, HFL1 cells	(76)
A Large Randomized Trial: Effects of Mindfulness-Based Stress Reduction (MBSR) for Breast Cancer (BC) Survivors on Salivary Cortisol and IL-6	randomized trial	IL-6, salivary cortisol	(77)
The Influence of Single Nucleotide Polymorphisms and Adjuvant Radiotherapy on Systemic Inflammatory Proteins, Chemokines and Cytokines of Patients With Breast Cancer	clinical trial	CRP, CCL4, IL-2, CRP, CCL5	(78)
Transcriptomic profiles conducive to immune-mediated tumor rejection in human breast cancer skin metastases treated with Imiquimod	clinical trial	Th-1	(79)
T follicular regulatory cells suppress Tfh-mediated B cell help and synergistically increase IL-10-producing B cells in breast carcinoma	clinical trial	Tfr-like, Treg-like, IL-10	(80)
Low Plasma IL-8 Levels During Chemotherapy Are Predictive of Excellent Long-Term Survival in Metastatic Breast Cancer	prospective phase 2 trial	IL-8	(81)
Cyclin E1 Expression and Palbociclib Efficacy in Previously Treated Hormone Receptor-Positive Metastatic Breast Cancer	clinical trial	CDK4, CDK6, cyclin D1, RB1	(82)
Dose-dependent effect of aerobic exercise on inflammatory biomarkers in a randomized controlled trial of women at high risk of breast cancer	randomized controlled trial	CCL-2, IL-12, TNF-alpha; IL-10	(83)
Phase I dose-escalation trial to repurpose propagermanium, an oral CCL2 inhibitor, in patients with breast cancer	multi-institutional, open-label, phase I study	CCL2, IL-6	(84)
Autologous dendritic cells and activated cytotoxic T-cells as combination therapy for breast cancer	phase I/II prospective study	CD19+ B cells, CD16+/CD56+	(85)
Efficacy and safety of the therapeutic cancer vaccine tecemotide (L-BLP25) in early breast cancer: Results from a prospective, randomized, neoadjuvant phase II study (ABCSG 34)	prospective, randomized, multicenter, phase II trial	MUC-1, RCB 0/I	(86)
Immunogenomic profiling and pathological response results from a clinical trial of docetaxel and carboplatin in triple-negative breast cancer	clinical trial	IDO-1, PD-L1, interferon gamma signaling, CTLA4, cytotoxicity, tumor inflammation signature, inflammatory chemokines, cytotoxic cells, lymphoid, PD-L2, exhausted CD8, Tregs, and immunoproteasome	(87)
Effects of an Exercise and Nutritional Intervention on Circulating Biomarkers and Metabolomic Profiling During Adjuvant Treatment for Localized Breast Cancer: Results From the PASAPAS Feasibility Randomized Controlled Trial	randomized controlled trial	insulin, insulin-like growth factor I, estradiol, adiponectin, leptin, IL-6, TNF alpha	(88)
Inflammation Mediates Exercise Effects on Fatigue in Patients with Breast Cancer	randomized controlled trial	IL-6, CD8a	(89)
Effects of Ketogenic metabolic therapy on patients with breast cancer: A randomized controlled clinical trial	randomized controlled trial	TNF-alpha, serum insulin	(90)
Biomarkers of response to Camrelizumab combined with apatinib: an analysis from a phase II trial in advanced triple-negative breast cancer patients	open-label, randomized, parallel, non-comparative, two-arms, phase II trial	TILs, CD8+, TIM-3, CD152, CD4+; HGF/IL-8	(91)
Phase II clinical trial using anti-CD3 × anti-HER2 bispecific antibody armed activated T cells (HER2 BATs) consolidation therapy for HER2 negative (0-2+) metastatic breast cancer	phase II clinical trials	interferon-γ immunospots, Th1 cytokines, Th2 cytokines, and chemokines	(92)
Effects of synbiotic supplementation on serum adiponectin and inflammation status of overweight and obese breast cancer survivors: a randomized, triple-blind, placebo-controlled trial	randomized, triple-blind, placebo-controlled trial	Adiponectin; TNF-alpha, hs-CRP	(93)
Resistance Exercise Modulates Kynurenine Pathway in Pancreatic Cancer Patients	randomized clinical trial	Serum kynurenine, kynurenine/tryptophan ratio, IL-6; tryptophan	(94)
The Impact of Preoperative Enteral Nutrition Enriched with Eicosapentaenoic Acid on Postoperative Hypercytokinemia after Pancreatoduodenectomy: The Results of a Double-Blinded Randomized Controlled Trial	double-blind randomized controlled trial	Il-6, IL-1beta, TNF-alpha, CD4/8	(95)

NA, (not applicable).

### 3.2 Biomarkers for breast cancer diagnosis and treatment

The most frequently measured biomarkers in breast cancer came from the interleukin family, with IL-6 being the most prevalent (**Table 3**). Other interleukin cytokines such as IL-1α, IL-1β, IL-2, IL-8, IL-10, and IL-12 were mentioned more than two times among 54 studies. TNF-α was also one of the most commonly reported biomarkers in breast cancer trials, and nine different studies discussed its use as an indication with clinical relevance. Moreover, many studies also focused on the use of cluster of differentiation (CD) molecules as an indication for breast cancer diagnosis and treatment. Cluster of differentiation (CD), also referred to as classification determinant or cluster of designation, refers to the methods used to identify and study the class of membrane surface proteins that serve as targets during the process for cellular immunophenotyping (96). They are involved in a multiplicity of cell signaling activities driving and responding to immune system perturbations, typically playing the role as either receptor or ligand (97), and they are often implicated in dysfunctional inflammatory mechanisms that result in disease outcomes. The most common CD molecules mentioned in the publications included in this review of breast and pancreatic cancers were CD4 and CD8, in the context of treatment (53, 55, 91); while others such as CD19 and CD16 were also assessed in several studies, both for treatment as well (85). In addition, CA15-3, Type 1 T helper cells (Th1), C-reactive protein (CRP), and VEGF were also used in more than three studies for breast cancer for the purpose of diagnosis and treatment (30, 33, 51, 54, 59, 61, 63, 66, 78, 79). Other biomarkers like insulin, C-C motif chemokine ligand 2 (CCL2), transforming growth factor β 1(TGF-β1), and Treg-like molecule (Treg: Regulatory T cells) were also discussed in some of our reviewed publications, regarding their relevance primarily to breast cancer treatment (75, 76, 80, 83, 84, 88, 90).

**Table 3:** Biomarker changes specific to breast cancer.

Name of Study	Biomarker Changes	Clinical Practice	Obesity Influence	Ref
The tumor-targeting immunocytokine F16-IL2 in combination with doxorubicin: dose escalation in patients with advanced solid tumors and expansion into patients with metastatic breast cancer	F16-IL2 fusion protein plus doxorubicin regimen was evaluated for safety, tolerability and activity, found no anti-fusion protein antibodies, and improvement in disease control rates of 57% and 67% at 8 weeks down to 43% and 33% after 12 weeks for Phase I and II study patients, respectively	treatment	NA	(26)
Improved Natural Killer cell activity and retained anti-tumor CD8(+) T cell responses contribute to the induction of a pathological complete response in HER2-positive breast cancer patients undergoing neoadjuvant chemotherapy	significant increase in HER2-positive patients	treatment	NA	(27)
High-circulating Tie2 Is Associated With Pathologic Complete Response to Chemotherapy and Antiangiogenic Therapy in Breast Cancer	associated with pCR	treatment	NA	(28)
Mindfulness and its efficacy for psychological and biological responses in women with breast cancer	increase within MBSR group, decrease between MBSP and non-MBSR	treatment	NA	(29)
Binding of circulating anti-MUC1 antibody and serum MUC1 antigen in stage IV breast cancer	there was a negative correlation between anti-MUC1 IgG and CA15-3 antigen in stage IV breast cancer when positive CA15-3 antigen and/or anti-MUC1 IgG were selected (r=−0.417; P=0.0044). The positive anti-MUC1 IgG with positive Ca15-3 antigen was more common in stage IV breast cancer, compared with early-stage breast cancer (χ2 = 4.629; P=0.031), however, Ca15-3 antigen positivity was higher in stage IV breast cancer, compared with early-stage breast	NA	NA	(30)
Receptor activator of nuclear factor kappa B (RANK) expression in primary breast cancer correlates with recurrence-free survival and development of bone metastases in I-SPY1 (CALGB 150007/150012; ACRIN 6657)	increased in HR negative patient	diagnosis	No	(31)
Dynamic changes of Receptor activator of nuclear factor-κB expression in Circulating Tumor Cells during Denosumab predict treatment effectiveness in Metastatic Breast Cancer	has association	treatment	NA	(32)
Effects of Molecular Iodine/Chemotherapy in the Immune Component of Breast Cancer Tumoral Microenvironment	increases	treatment	NA	(33)
Efficacy and Determinants of Response to HER Kinase Inhibition in HER2-Mutant Metastatic Breast Cancer	HER2 mutation increases	treatment	NA	(34)
Intratumoral Hydrogen Peroxide With Radiation Therapy in Locally Advanced Breast Cancer: Results From a Phase 1 Clinical Trial	decrease	treatment	NA	(35)
Effects of Fresh Yellow Onion Consumption on CEA, CA125 and Hepatic Enzymes in Breast Cancer Patients: A Double-Blind Randomized Controlled Clinical Trial	increased	treatment	NA	(50)
Targeted T-cell Therapy in Stage IV Breast Cancer: A Phase I Clinical Trial	increase	treatment	NA	(51)
Inflammation and psychosocial factors mediate exercise effects on sleep quality in breast cancer survivors: pilot randomized controlled trial	significant difference between groups	treatment	NA	(52)
ELYPSE-7: a randomized placebo-controlled phase IIa trial with CYT107 exploring the restoration of CD4+ lymphocyte count in lymphopenic metastatic breast cancer patients	increase	treatment	NA	(53)
A Comparison of Fentanyl and Flurbiprofen Axetil on Serum VEGF-C, TNF-α, and IL-1ß Concentrations in Women Undergoing Surgery for Breast Cancer	higher in Group F	treatment	NA	(54)
Effect of Propofol and Desflurane on Immune Cell Populations in Breast Cancer Patients: A Randomized Trial	preserved	treatment	NA	(55)
Postsurgical Depressive Symptoms and Proinflammatory Cytokine Elevations in Women Undergoing Primary Treatment for Breast Cancer	higher in participants with higher depression level	NA	NA	(56)
Association of CA27.29 and Circulating Tumor Cells Before and at Different Times After Adjuvant Chemotherapy in Patients with Early-stage Breast Cancer - The SUCCESS Trial	NA	treatment	NA	(57)
Resistance Exercise and Inflammation in Breast Cancer Patients Undergoing Adjuvant Radiation Therapy: Mediation Analysis From a Randomized, Controlled Intervention Trial	increased	treatment	NA	(58)
Angiogenic cytokines and their influence on circulating tumour cells in sera of patients with the primary diagnosis of breast cancer before treatment	increased in CTC-negative patients	diagnosis	NA	(59)
Relationship of inflammatory profile of elderly patients serum and senescence-associated secretory phenotype with human breast cancer cells proliferation: Role of IL6/IL8 ratio	IL-6/IL-8 ratio more than 2.0 can induce cellular proliferation of MCF-7	NA	NA	(60)
Testing breast cancer serum biomarkers for early detection and prognosis in pre-diagnosis samples	increased	diagnosis	NA	(61)
Tumor cryoablation in combination with natural killer cells therapy and Herceptin in patients with HER2-overexpressing recurrent breast cancer	reduced	treatment	NA	(62)
An anti-inflammatory dietary intervention to reduce breast cancer recurrence risk: Study design and baseline data	positive relationship with BMI	treatment	Yes	(63)
Association between changes in fat distribution and biomarkers for breast cancer	decrease	treatment	Yes	(64)
combined levels of MMP-1, hepatocyte growth factor, and chemokine ligand 5 used as a three markers-score model, to predict axillary lymph node metastasis positivity	three marker scores can indicate positive lymph node metastasis	diagnosis	No	(65)
the effect analysis of CYP2D6 gene polymorphism in the toremifene and tamoxifen treatment in patient with breast cancer	lower after operation	treatment	NA	(66)
Increased interleukin-35 expression in tumor-infiltrating lymphocytes correlates with poor prognosis in patients with breast cancer	increase	diagnosis	NA	(67)
Adipose tissue inflammation in breast cancer survivors: effects of a 16-week combined aerobic and resistance exercise training intervention	decreased	treatment	Yes	(68)
Benefits in radical mastectomy protocol: a randomized trial evaluating the use of regional anesthesia	no differences between groups	NA	NA	(69)
Vitamin D Levels, Vitamin D Receptor Polymorphisms, and Inflammatory Cytokines in Aromatase Inhibitor-Induced Arthralgias: An Analysis of CCTG MA.27	IL-1beta decreased	NA	NA	(70)
Prognostic impact of CD4-positive T cell subsets in early breast cancer: a study based on the FinHer trial patient population	high CXCL 13 was associated with favorable distant disease-free trial	treatment	NA	(71)
IL1 Receptor Antagonist Controls Transcriptional Signature of Inflammation in Patients with Metastatic Breast Cancer	production of IL1 β in primary breast cancer tumors is linked with advanced disease and originates from tumor-infiltrating CD11c+ myeloid cells	treatment	NA	(72)
Reducing postsurgical exudate in breast cancer patients by using San Huang decoction to ameliorate inflammatory status: a prospective clinical trial	decreased with SHD treatment	treatment	NA	(73)
Atezolizumab Plus nab-Paclitaxel in the Treatment of Metastatic Triple-Negative Breast Cancer With 2-Year Survival Follow-up: A Phase 1b Clinical Trial	not significantly changed	treatment	NA	(74)
NSAID treatment reduced transcript expression of MMPs, CCL22 and TGF-b1, which translated to reduction in metastasis-promoting cell population	reduction in MMPs, CCL22 and TGF-β1 expression reduces tumor renewal and metastasis with NSAID treatment	treatment	NA	(75)
Sapylin (OK-432) alters inflammation and angiogenesis *in vivo* and *vitro*	increased/enhanced	treatment	NA	(76)
A Large Randomized Trial: Effects of Mindfulness-Based Stress Reduction (MBSR) for Breast Cancer (BC) Survivors on Salivary Cortisol and IL-6	reduced	treatment	NA	(77)
The Influence of Single Nucleotide Polymorphisms and Adjuvant Radiotherapy on Systemic Inflammatory Proteins, Chemokines and Cytokines of Patients With Breast Cancer	dysregulation in breast cancer patient	treatment	NA	(78)
Transcriptomic profiles conducive to immune-mediated tumor rejection in human breast cancer skin metastases treated with Imiquimod	upregulated	treatment	NA	(79)
T follicular regulatory cells suppress Tfh-mediated B cell help and synergistically increase IL-10-producing B cells in breast carcinoma	elevated	diagnosis	NA	(80)
Low Plasma IL-8 Levels During Chemotherapy Are Predictive of Excellent Long-Term Survival in Metastatic Breast Cancer	decreased in trajectory group 1	treatment	NA	(81)
Cyclin E1 Expression and Palbociclib Efficacy in Previously Treated Hormone Receptor-Positive Metastatic Breast Cancer	no significant interaction	treatment	NA	(82)
Dose-dependent effect of aerobic exercise on inflammatory biomarkers in a randomized controlled trial of women at high risk of breast cancer	linear dose-response relationship	prevention	NA	(83)
Phase I dose-escalation trial to repurpose propagermanium, an oral CCL2 inhibitor, in patients with breast cancer	decreased	treatment	NA	(84)
Autologous dendritic cells and activated cytotoxic T-cells as combination therapy for breast cancer	increased	treatment	NA	(85)
Efficacy and safety of the therapeutic cancer vaccine tecemotide (L-BLP25) in early breast cancer: Results from a prospective, randomized, neoadjuvant phase II study (ABCSG 34)	no significant differences	treatment	NA	(86)
Immunogenomic profiling and pathological response results from a clinical trial of docetaxel and carboplatin in triple-negative breast cancer	upregulated	treatment	NA	(87)
Effects of an Exercise and Nutritional Intervention on Circulating Biomarkers and Metabolomic Profiling During Adjuvant Treatment for Localized Breast Cancer: Results From the PASAPAS Feasibility Randomized Controlled Trial	no significant changes	treatment	NA	(88)
Inflammation Mediates Exercise Effects on Fatigue in Patients with Breast Cancer	less pronounced after RT-HIlT	treatment	NA	(89)
Effects of Ketogenic metabolic therapy on patients with breast cancer: A randomized controlled clinical trial	decreased	treatment	NA	(90)
Biomarkers of response to camrelizumab combined with apatinib: an analysis from a phase II trial in advanced triple-negative breast cancer patients	increase/higher	treatment	NA	(91)
Phase II clinical trial using anti-CD3 × anti-HER2 bispecific antibody armed activated T cells (HER2 BATs) consolidation therapy for HER2 negative (0-2+) metastatic breast cancer	increases	treatment	NA	(92)
Effects of synbiotic supplementation on serum adiponectin and inflammation status of overweight and obese breast cancer survivors: a randomized, triple-blind, placebo-controlled trial	increased	treatment	Yes	(93)

NA, (Not Applicable) and BMI, (Body Mass Index).

#### 3.2.1 IL-6

IL-6 was the most frequently mentioned cytokine across all breast cancer studies included in this review. IL-6 is part of the interleukin superfamily, which includes more than 50 molecules with various kinds of functions such as maturation, proliferation, inflammation, and differentiation (98, 99). IL-6, like several cytokines (i.e., hepatocyte growth factor (HGF) (100) is pleiotropic and acts as both a pro-inflammatory and anti-inflammatory cytokine; thus, the levels of changes and activity across our review were inconsistent. Some studies showed a significant decrease in IL-6 levels after treatment or specific interventions, including exercise, San Huang decoction, Mindfulness-Based Stress Reduction (MBSR), and use of the C-C motif chemokine receptor 2 (CCR2) inhibitor (101) propagermanium (68, 73, 77, 84, 89). Both Hiensch et al. and Dieli-Conwright et al. explained the effect of exercise (HIIT – high impact interval training and general aerobic and resistance exercise) on reducing IL-6 level in breast cancer patients and survivors. In a pilot randomized controlled trial with 46 postmenopausal breast cancer survivors, the 3-month exercise intervention resulted in a significant difference in IL-6 between the intervention and control group (52). On the other hand, some investigations have demonstrated an increase in IL-6 following breast cancer treatment (58, 76), plausibly due to a normal inflammatory response as a result of introducing chemo-toxicants into the body. One study finding worth noting was according to the investigation by Schmidt et al. in a randomized controlled trial examining the relationship between resistance exercise and inflammation in 103 breast cancer patients with radiation therapy, that mentioned the effect of only this type of exercise, which significantly increased the level of IL-6 (58). Moreover, another study focused on the effect of an anti-inflammatory diet, revealed a positive relationship between IL-6 and BMI in breast cancer patients (63). In a few of the studies reviewed here-in, there was no difference or IL-6 level change following their respective interventions (69, 70, 88). Other note-worthy findings relative to breast cancer did include those found by Matsumoto et al. where the use of regional anesthesia did not influence the level of IL-6 and by Niravath et al. showing no significant effect of vitamin D nor vitamin D receptors on the inflammatory cytokines they monitored (69, 70). Surprisingly though and likewise, in the PASAPAS Feasibility Randomized Controlled Trial of 61 women given adjuvant chemotherapy, aerobic exercise did not make any notable effect on circulating levels of seven different biomarkers assessed, including IL-6 which was also monitored in this study (88).

#### 3.2.2 TNF-alpha (α)

TNF-α was the second most frequently inflammatory cytokine mentioned by studies in this review related to breast cancer. In the 10 studies that measured the level of TNF-α, six of them concluded that a lower level of the marker can indicate a better disease prognosis or outcome with treatment (54, 56, 63, 68, 73, 90); while two found no notable changes (70, 88), one showed an increased level of change related to moderate-to-vigorous aerobic exercise (83), and the other found that TNF-α can be a mediator for sleep response following exercise intervention in breast cancer patients (52). Although most results show a benefit when it comes to the influence of exercise on inflammation related biomarkers, there is still some controversy. For example, according to Dieli-Conwright et. al., a 16-week aerobic and resistance exercise intervention decreased TNF-α among obese postmenopausal breast cancer survivors (68). However, the study performed by Haley and colleagues demonstrated that moderate to vigorous exercise increased the level of several pro-inflammatory biomarkers, including TNF-α, albeit in healthy premenopausal women; and the mechanism of reducing risk may not involve inflammatory molecules (83).

#### 3.2.3 IL-1 beta (β)

Unlike IL-6, IL-1β had more consistent results across our reviewed studies, with the exception of the results from one study conducted by Matsumoto et. al (69). In this single-center, prospective, randomized clinical trial, the use of regional anesthesia had no significant effect on changing biomarker levels (69), whereas overall, results from various other studies indicated that a lower level of IL-1β was associated with better breast cancer outcomes. An example includes a 13-participant clinical trial, where patients were treated with intertumoral H_2_O_2_ and with RT, as the treatment showed an effect on decreasing IL-1β (35). Moreover, in another study including 40 women with primary breast cancer undergoing - modified radical mastectomy, the use of postoperative analgesia with flurbiprofen axetil, combined with fentanyl, also decreased the level of IL-β (54). In the case-control study focused on the effect of vitamin D, although vitamin D levels were not significantly associated with the development of Aromatase inhibitor-induced Arthralgias (AIA), patients with the Fok-I Vitamin D receptor (VDR) variant genotype were more likely to experience a reduction in IL-1β among other inflammatory cytokines (70). Most importantly from our studies, the production of IL-1β was associated more often with advanced breast cancer, as well as a higher level of depression, evidenced by two different clinical trials, respectively (56, 72).

#### 3.2.4 VEGF

Vascular endothelial growth factor (VEGF) is a potent angiogenic factor in both healthy people and cancer patients, which is often upregulated in many tumor types (102). In healthy people, increases in VEGF promotes embryonic development and wound healing through angiogenesis (103). However, in cancer patients, the production of VEGF and other growth factors promotes new blood vessel formation around cancer cells and throughout tumors supplying both nutrients and oxygen (103). This characteristic of VEGF makes it one of the most widely studied targets for anticancer therapeutic development and its activity is intricately linked to evaluating drug treatment efficacy. In this review, four separate studies measured the level change of VEGF in breast cancer related to an intervention/treatment. In the study by Wen et. al., that measured the effect of postoperative analgesia using flurbiprofen axetil combined with fentanyl, patients’ levels of VEGF decreased compared with patients receiving only fentanyl (54). Moreover, in comparing 100 Circulating Tumor Cells (CTC) negative and 100 CTC positive patients with breast cancer, researchers found that VEGF increased in CTC-negative patients, suggesting that it could serve as a potential biomarker (59). Also, investigating the treatment of toremifene and tamoxifen demonstrated a decrease in the level of VEGF in patients with ER-positive breast cancer plus a (Cytochrome P450) CYP2D6 gene polymorphism (66). This study by Zeng et. al., particularly reiterates the importance of evaluating potential and validated biomarkers in the context of genetic factors and driver mutations that influence the overall nature of the malignancy. Single nucleotide polymorphisms especially can impact treatment efficacy and in-turn change the activity or expression level of many cytokines. Sapylin (OK-432) is a drug derived from the group A hemolytic *streptococcus Su* strain, which can help reduce seroma drainage and promote wound healing (76). In the prospective consecutive cohort study that analyzed the effect of Sapylin on breast cancer patients that had undergone radical mastectomy, VEGF was increased in the drainage fluids of patients treated with Sapylin, and these patients had a better wound healing response (76). This might be an indication for patients that have larger areas of normal tissue around the tumor removed with the tumor, using drugs that increase VEGF production, such as Sapylin, will benefit the overall survival and outcome. Using such drugs to augment improved treatment response following surgery, which itself increases tissue inflammation (104), clearly need further consideration and validation in all cancer types where surgery is part of the primary treatment plan.

#### 3.2.5 CA15-3

CA15-3 is another protein like VEGF, expressed by various cell types, including breast cancer cells. It is a substance that stimulates the body’s defense system, which makes it a useful biomarker for detecting the presence of disease, diagnosing stage, and determining optimal treatment regimen for breast cancer. Four studies here, also measured changes of CA15-3 among all the included breast cancer articles in this review. In a study analyzing serum samples in 61 patients with stage IV breast cancer and 64 patients with early-stage breast cancer, researchers found a higher CA15-3 positivity level in stage IV breast cancer patients than in early-stage patients, which clearly showed that CA15-3 could reflect the progression of breast cancer (30). For treatment, Liang et. al., showed that the three drug-therapy combination of cryoablation, natural killer (NK) cells, and Herceptin effectively reduced the level of CA15-3 and improved breast cancer outcomes (62). However, in Kazarian et. al., researchers also mentioned that although CA15-3 has the potential to be a prognostic marker for breast cancer, further research is needed because there were no significant findings on the function of CA15-3 in breast cancer screening (61).

#### 3.2.6 IDO-1

Indoleamine-pyrrole 2,3-dioxygenase (IDO-1) is a heme-containing enzyme, which acts as a rate-limiting enzyme in tryptophan metabolism (105). IDO-1 appeared only once among our included breast cancer articles, in a study where researchers analyzed the immunogenomic profiling and pathological response to neoadjuvant therapy including docetaxel and carboplatin, both commonly used in the treatment for breast cancer. The results showed that IDO-1, in addition with other immunoproteasome mediators were upregulated before treatment was administered at baseline (87). Thus, the treatment with docetaxel and carboplatin potentially decreased the level of IDO-1 and may improve disease outcome. As such, IDO-1 could be considered as an important emerging biomarker related to breast cancer treatment, and especially in relation to immune-based therapy.

#### 3.2.7 IL-8

IL-8 is also one of frequently measured inflammatory biomarkers in breast cancer studies. In five studies that measured IL-8 levels, three of them indicated that a lower level of IL-8 is associated with better overall survival (73, 81, 91). In Liu et. al, patients with lower baseline plasma levels of IL-8 were also found to more likely respond to a combination treatment of monoclonal antibodies camrelizumab and apatinib and showed a longer overall survival (91). Moreover, a similar finding was shown in Tiainen et al. as well that lower plasma IL-8 during chemotherapy can predict excellent long-term survival (81). In an analysis using a traditional Chinese combination of three “cooling” herbs, San Huang decoction, known to effectively alleviate injury-related pain and inflammation, researchers found that the level of IL-8 was significantly lower following treatment, as one of the signs of decreased inflammation status in breast cancer patients (73). In the other two studies, one confirmed a positive relationship between BMI and IL-8, but did not detail how the relationship could impact disease outcomes (63); while the other study explored whether inflammatory factors could mediate exercise effects on sleep quality for breast cancer survivors, but failed to find any significant impact of IL-8 levels, concluding this marker alone was not sufficient to result in sleep-associated outcomes (52).

#### 3.2.8 Natural killer cells

Natural Killer cells (NK cells) are one type of immune cells that can control several types of tumors and microbial infection (106). Based on the articles that were included in this review, the results regarding the clinical utility of NK cells as a biomarker for breast cancer are consistent. For example, in the study by Tsukinaga and colleagues, who analyzed the efficacy of a combination treatment of F16-IL2 (a recombinant antibody-cytokine fusion protein) and doxorubicin in patients with solid tumors and metastatic breast cancer, NK cells were increased following treatment, which improved overall cellular immunity (37). In addition, in exploring the effect of mindfulness on psychological and biological responses in women with breast cancer, researchers found that NK cell activity increased significantly within the mindfulness-based stress reduction group. Overall, according to the reviewed studies, an increase of NK cells within the microenvironment and systemically indicates better immunity, which presumably contributes to better disease outcome (27, 29, 62).

#### 3.2.9 C-reactive protein

It stands to reason that the general serum inflammation marker CRP or highly sensitive-CRP (hs-CRP), which is sometimes measured, has high clinical relevance because it is easy to measure and it has long-been used in most clinical settings to assess inflammation status related to chronic diseases, as well as acute inflammation. C-reactive protein is a protein made by the liver and increases when there is inflammation present. In terms of cancer, the marker is clinically relevant because it has been shown that if it can be lowered, it serves as a readout of lower systemic or local inflammation and can foster improved cancer outcomes through a host of immune mechanisms. In our review, four articles that demonstrated clinical utility under the breast cancer selection were included. Two of which indicated that better disease outcomes could be achieved by certain treatments, including adjuvant radiotherapy (78), where there was a reduction in the level of CRP. The other study, conducted by Lahiji and colleagues, was an interesting randomized, triple-blind, placebo-controlled trial using a low calorie diet in obese breast cancer survivors who either received synbiotic supplementation (the combination of probiotics and prebiotics) or not and found hs-CRP as well as adiponectin levels can be lowered and likely indicate reduced rates of recurrence (93). Further, in another study analyzing the relationship between fat distribution and level of biomarkers in breast cancer showed that changes in abdominal fat were associated with changes in CRP levels (64), while the final reviewed study showed a positive relation between BMI and CRP (63).

### 3.3 Biomarkers for pancreatic cancer diagnosis and treatment

All pancreatic cancer-specific biomarker changes are summarized in **Table 4**. The most frequently mentioned biomarkers for this cancer type came from the interleukin superfamily including IL-6, IL-8, IL-10, IL-2, IL-18, which is quite similar to what was found in breast cancer. Moreover, CD molecules related to T cells, including CD4+ and CD8+ T cells were mentioned more often in pancreatic than in breast cancer studies. Finally, the CXC motif chemokine ligand (CXCL) and CCL family of markers were mentioned more than two times for different molecules. For the CXCL family, CXCL 9 and CXCL 10 were both mentioned once, respectively, and likewise for CCL2, CCL5, and CCL27 in the CCL family. Since the overall pool of included articles for pancreatic cancer-related inflammation biomarkers is much smaller compared to number for breast cancer, the total number of biomarkers mentioned is also less extensive. Even-so, we discuss some that are common to both cancers, as well as those that are unique to pancreatic cancer according to our systematic criteria.

**Table 4:** Biomarker changes specific to pancreatic cancer.

Name of Study	Biomarker changes	Clinical Practice	Obesity Influence	Ref
Prognostic and predictive value of immunological parameters for chemoradioimmunotherapy in patients with pancreatic adenocarcinoma	high lymphocyte accumulation in tumors and frequencies of NK cells and effector (eff) CD8 T cells in peripheral blood were associated with patients’ survival. Amount of CD3 and effector-memory CD8 blood lymphocytes, expression of CD152 and interleukin (IL)-2 serum level showed a predictive value for chemoradioimmunotherapy. Tumoral accumulation of CD3 and CD8 cells was predictive for outcome of chemotherapy alone. Frequencies of CD3 lymphocytes, effCD8 T cells and NK cells in peripheral blood of patients, and IL-10 amount in serum, was predictive for 5FU-based chemotherapy.	both	NA	(24)
BL-8040, a CXCR4 antagonist, in combination with pembrolizumab and chemotherapy for pancreatic cancer: the COMBAT trial	increased	treatment	NA	(25)
Phase I pilot study of Wilms tumor gene 1 peptide-pulsed dendritic cell vaccination combined with gemcitabine in pancreatic cancer	increase in patient without liver metastases		NA	(36)
Prognostic significance of plasma interleukin-6/-8 in pancreatic cancer patients receiving chemoimmunotherapy	decreased	treatment	NA	(37)
Phase II trial of salvage therapy with trabectedin in metastatic pancreatic adenocarcinoma	significantly reduced with treatment	treatment	NA	(38)
Systemic immune activity predicts overall survival in treatment naive patients with metastatic pancreatic cancer	poorer overall survival	diagnosis	NA	(39)
Immunobiological effects of gemcitabine and capecitabine combination chemotherapy in advanced pancreatic ductal adenocarcinoma	no differences	treatment	NA	(40)
Randomized Phase 2 Trial of the Oncolytic Virus Pelareorep (Reolysin) in Upfront Treatment of Metastatic Pancreatic Adenocarcinoma	increased	treatment	NA	(41)
Phase 1b study targeting tumour associated macrophages with CCR2 inhibition plus FOLFIRINOX in locally advanced and borderline resectable pancreatic cancer	decreased in both bone marrow and peripheral blood	treatment	NA	(42)
Pharmacogenomic analyses of sunitinib in patients with pancreatic neuroendocrine tumors	significantly associated with higher ORR (G/A)	treatment	NA	(8)
Phase I clinical trial repurposing all-trans retinoic acid as a stromal targeting agent for pancreatic cancer	increased stomal expression related to better disease control	treatment	NA	(43)
Plasma IL8 Is a Biomarker for TAK1 Activation and Predicts Resistance to Nanoliposomal Irinotecan in Patients with Gemcitabine-Refractory Pancreatic Cancer	decreased	treatment	NA	(44)
Phase 1b study of a small molecule antagonist of human chemokine (C-C motif) receptor 2 (PF-04136309) in combination with nab-paclitaxel/gemcitabine in first-line treatment of metastatic pancreatic ductal adenocarcinoma	increased in most patient	treatment	NA	(45)
A phase 1b trial of concurrent immunotherapy and irreversible electroporation in the treatment of locally advanced pancreatic adenocarcinoma	increased	treatment	NA	(46)
Randomized Phase III Study of FOLFOX Alone or With Pegilodecakin as Second-Line Therapy in Patients With Metastatic Pancreatic Cancer That Progressed After Gemcitabine (SEQUOIA)	increased	treatment	NA	(47)
Immunologic and tumor responses of pegilodecakin with 5-FU/LV and oxaliplatin (FOLFOX) in pancreatic ductal adenocarcinoma (PDAC)	elevated	treatment	NA	(48)
Circulating biomarkers and outcomes from a randomized phase 2 trial of gemcitabine versus capecitabine-based chemoradiotherapy for pancreatic cancer	patients with high circulating CCL5 were found to have a HR of 1.01 for each ng/ml unit increase	treatment	NA	(49)
Resistance Exercise Modulates Kynurenine Pathway in Pancreatic Cancer Patients	decreased	treatment	NA	(94)
The Impact of Preoperative Enteral Nutrition Enriched with Eicosapentaenoic Acid on Postoperative Hypercytokinemia after Pancreatoduodenectomy: The Results of a Double-Blinded Randomized Controlled Trial	no differences between groups	treatment	NA	(95)

NA, (Not Applicable); ORR, (Objective Response Rate); HR, (Hazard Ratio).

#### 3.3.1 IL-6

Again, for pancreatic cancer, IL-6 was the biomarker mentioned most frequently. Among the 19 pancreatic cancer papers included in this review, IL-6 was mentioned in seven. Although the results and indications of IL-6 were still inconsistent, more studies showed that a lower IL-6 level indicated better disease outcomes and more effective treatments. In studies that measured the effect of chemo-immunotherapy, salvage therapy, and supervised resistance exercise, all researchers noticed a significant decrease in IL-6 levels (37, 38, 94). Moreover, the study by Farren et. al., analyzing peripheral blood from 73 pancreatic cancer patients explained that having a higher IL-6 level predicted a poorer overall survival (39). Despite four studies demonstrating results of a lower IL-6 level as better (37–39, 94), two additional studies found no differences, where one measured the effect of a combination of gemcitabine and chemotherapy (40), and the other investigated a pre-operative immuno-nutrition intervention (95). The only study that explored an increase in IL-6 level as one of the outcomes, was testing the function and safety of Pelareorep as a treatment for pancreatic cancer (41).

#### 3.3.2 CD8+ T cells

CD8+ T cells are among an important group of molecules in the MHC class I-restricted T cells, and they function as a part of our essential immune defense against intracellular pathogens and for tumor cell surveillance. Several studies detailed changes of CD8+ T cells, and three of which showed increased results. The accumulation of CD8+ T cells can be a good predictor of patients’ survival and outcome with chemotherapy (24), and the combined use of CXCR 4 and PD-1 blockade was also shown to expand the benefit of chemotherapy, through a mechanism that increased CXCR-expressing CD8+ T cell levels (25). Moreover, in Noonan et. al., Pelareorep both increased IL-6 levels but also upregulated CD8+ T cells, resulting in more cytotoxic T-lymphocyte associated protein 4 (CTLA4), a protein found on T cells (a type of immune cell) that helps keep the body’s immune responses in check (41). In contrast, Farren et al. pointed out that having a higher CTLA4 on CD8+ T cells at the baseline (before treatment) predicted a poorer overall survival for pancreatic cancer patients (39).

#### 3.3.3 CA19

CA19 is a tetra-saccharide which is often found attached to O-glycans on the surface of several types of cells, and it is usually a specific marker for identifying the presence of pancreatic cancer since ductal cells in the pancreas can produce it. CA19 was mentioned twice among the 19 reviewed pancreatic cancer trials. In Karakhanova et. al., a clinical trial that identified immunological parameters in the treatment and prognosis of pancreatic carcinoma patients, CA19 was determined to be a potential predictive marker of treatment efficacy for both chemo-radioimmunotherapy and chemotherapy (24). Moreover, in a multi-institutional, open-label, multiple-cohort, dose-escalation, phase 1b study that analyzed the effect of using pegilodecakin with 5-FU/LV and oxaliplatin (FOLFOX), about 66.7% of measurable patients showed a decline in CA-19 as a response (48).

#### 3.3.4 IDO-1

Similarly, as in the publications for breast cancer, IDO-1 was also discussed in only one of the pancreatic cancer trials reviewed here-in. The primary difference is that the researchers for the pancreatic study did not measure it directly, as was performed in the breast cancer study. This study, conducted by Pal and his colleagues, examined how resistance exercise modulates the kynurenine pathway in pancreatic cancer patients (94). Tryptophan feeds into the kynurenine pathways is typically disrupted when inflammation persists and can result in dysfunctional immune activation (107). Thus, the researchers found that a decreased kynurenine/tryptophan ratio, which suggests downregulated IDO levels, and their subsequent observation of lower levels of IDO, including IDO-1, links the marker to possible detection of reduced disease progression in pancreatic cancer patients (94).

#### 3.3.5 IL-8

Regarding the use of IL-8 level as a measurement for pancreatic cancer treatment, five studies in total all came to quite consistent results that a lower level of IL-8 indicated better survival (36–38, 44, 102). This conclusion makes results in pancreatic cancer consistent with most of the findings from studies of this inflammatory biomarker in breast cancer (73, 81, 91). For example, in the study that investigated the association between plasma IL-8 and clinical outcomes of chemoimmunotherapy in patients with pancreatic ductal adenocarcinoma, Tsukinaga et al. determined that having long-term low levels during chemo-immunotherapy may serve as a prognostic marker of clinical outcomes because elevated levels of circulating IL-8 have already been associated with experiencing a poorer outcome (37). In addition, Belli et al. concluded that lower IL-8 was significantly associated with overall survival, although their targeted therapy still showed no activity for metastatic pancreatic adenocarcinoma (38); representing a potential gap that could be informed by work done in breast cancer. In the study that used a mouse model co-trial design including patients with gemcitabine-refractory pancreatic cancer, to determine the effect of nano-liposomal irinotecan (nal-IRI), where a liposomal formulation of the topoisomerase I inhibitor serves as the drug delivery system, Merz et al. showed that treated mice with lower plasma levels of IL-8 also experienced a significant reduction in tumor growth (44). The other two studies showed the negative effect of high plasma IL-8, reinforcing the utility that lowering IL-8 has an implication for improved disease survival (36, 41).

#### 3.3.6 Natural killer cells

Although only one study measured and determined clinical relevance for the level of NK cells in pancreatic cancer, the indication was similar to that found by the publications looking at it in the context of breast cancer. Karakhanova et al. analyzed immunological aspects of pancreatic cancer patients undergoing chemo-radioimmunotherapy, and found that high NK cells level was associated with patient survival, concluding NK cells could serve as a useful marker for personalized medicine (24). Although studies are limited, based on this result, as well as supportive findings from several breast cancer-specific studies, future investigations to understand and further validate the mechanism(s) by which NK cells boost host immunity to properly act against tumor cells and maneuver around immuno-suppressive programs that other immune cell population succumb to in tumor microenvironments are greatly warranted. Clues from recent pre-clinical studies, for example, could be very helpful, including work in murine models showing how NK cells play a role in increasing tumoricidal CD8+ T-cell populations and suppressing PD-1 activity (108), can augment chimeric antigen receptor CAR T-cell therapies (109); or can activate mechanisms like SUMOylation to inhibit cell cycle of progression with novel drug therapies (110).

#### 3.3.7 C-reactive protein

Although CRP is a very common inflammation marker, in the context of our review, we only found two reported studies that fit our primary inclusion criteria of demonstrating usefulness in the clinic related to pancreatic cancer outcomes; and they had different results. In the first, Mayanagi et al. identified the effect of marker Wilms tumor gene 1 peptide-pulsed dendritic cell vaccination combined with gemcitabine showed that patients with liver metastases had higher levels of CRP (36). This result is similar to the study findings related to breast cancer, where higher CRP indicates a worse disease outcome. However, in the second study that determined the effect of a combination chemotherapeutic regimen containing gemcitabine and capecitabine, no significant differences were found in CRP level (40). More validation studies will be needed to confirm the relationship between certain treatment types and the utility of corresponding CRP level in pancreatic cancer.

## 4 Discussion

### 4.1 Implication for pancreatic cancer from breast cancer

#### 4.1.1 Genes and hormones

It is well established that BRCA gene mutations contribute to the increased risk of hereditary breast cancer (2, 111, 112). The prevention strategies (genetic screening) and therapies designed to target and diminish the actions of these genes, such as Olaparib, have been shown to be effective (113). These strategies can also be investigated for their utility as potential prevention and treatment methods for pancreatic cancer, especially in light of recent research implicating that BRCA gene mutations also plays an important role in increasing pancreatic cancer risks (12, 13). However, one thing that needs further consideration prior to implementation of any of these breast cancer-established strategies (preventative and therapeutic) is the fact that pancreatic cancer is not as easily detected as breast cancer. Most of the time, at the point which people are diagnosed with pancreatic cancer, they are at a later stage, their tumor is found in non-operable locations of the organ and often are of a histological type that do not respond as effectively to treatments. All these factors contribute to the much lower 5*-*year survival rate (11.5%) of pancreatic cancer compared to breast cancer (90.6%) (2, 8). Suppose for example, there is proof that a BRCA-targeted intervention could remain functional in later-stage breast cancer patients. In such a case, this intervention could also be a future method to explore for treating pancreatic cancer patients at similar times of later-stage diagnoses.

In addition to genetic aspects of cancer risk and treatment strategies, sex hormones such as estrogen and progesterone which have long held and continue to have an increasing interest in prevention, diagnosis and treatment for breast cancer research should also be given greater scrutiny for their role in pancreatic cancer. In the subtypes of breast cancer driven by estrogen – or the estrogen receptor-positive forms (114, 115), the expression and interaction of estrogen with the receptors causes these cancer cells to grow. Interestingly, in pancreatic cancer, estrogen has been shown to inhibit pancreatic cancer cell growth in experimental studies, because pancreatic cancer cells also express estrogen receptors (116). This finding is in stark contrast to the action of estrogen on breast cancer cells. So, based on the analysis of estrogen treatment in breast cancer, it is plausible that a targeted type of therapy could be effective in pancreatic cancer patients, but in the augmenting manner and especially for men, who are at lower risk for breast cancer development and complications in general, that would be due to elevated levels of circulating estrogen.

#### 4.1.2 Inflammatory biomarkers

This systematic review discussed current findings of inflammatory biomarkers, some common and some unique, identified in different trial designs of clinical studies from breast cancer and pancreatic cancer. Because both organs are largely regulated by the endocrine system, we hoped to determine if knowledge could be gained by the comparison, especially looking at breast cancer to inform new potential strategies for pancreatic cancer. After reviewing a total of 73 articles across both cancer types and based on our selection criteria, IL-6 was the most prevalent biomarker identified. Again, the results were variable, namely due to this myokine having both pro-inflammatory and anti-inflammatory properties. However, across the studies under each specific cancer type and when comparing the two cancers, there were some common trends (**Table 5**). For example, in both breast cancer and pancreatic cancer, many studies analyzed the effect of exercise, but again, the ultimate findings were inconsistent. For some patients, exercise could lower the level of inflammation (68, 89, 94), while for others, it did not (58, 83). The major differentiator we found was pre- versus postmenopausal status. Thus, exercise could be considered a potential personalized treatment as part of an integrated plan, particularly in the breast cancer setting, based on the patient’s age, disease stage, drug response, or even genetic test results that factor in inflammation at the outset based on defined markers like IL-6. Moreover, and uniquely in pancreatic cancer studies, research indicated that lower IL-6 might associate with a better disease outcome (39), where IL-6 clearly acts as a pro-inflammatory biomarker for this cancer type.

**Table 5:** Trial-defined inflammatory biomarker changes with clinical utility in both breast cancer and pancreatic cancer.

Biomarker	Biomarker changes in breast cancer	Biomarker changes in pancreatic cancer	Clinical Relevance/Overall Indication
CD4 T cells	CD4 level increases with CYT107 treatment (53). Having higher baseline CD4 may respond better for combinational anti-angiogenesis and immunotherapy in advanced triple-negative breast cancer (91). CD4 was not associated with distant disease-free survival (71).	High proportion of CD4 indicate longer overall survival (39). The accumulation of CD4+ cells correlated with survival benefits (24). CD4 level increased with BL-4080 treatment (25). CD4 increases with PF-04136309 treatment (42). Study demonstrated no difference (95).	Higher CD4 levels indicate better outcome/response to treatment in most studies.
CD8+T cells	Increased CD8 level with CYT107 treatment (53). Increase of tumor-infiltrating CD8+ T cell during therapy was associated with higher objective response rate (91). Increased CD8 level with neoadjuvant chemotherapy (27). No significant change (74).	CD8 level increases with Reolysin treatment (41). High CD8 associated with patients survival (24). CD8 level increases with BL-8040 treatment (25). Increased CD8 level with FOLFIRINOX treatment (42). No difference (95).	Higher CD8 level indicates better outcome, and several therapeutics demonstrated ability to increase the level of CD8 cells.
CRP	Adjuvant radiotherapy reduced plasma CRP (78). Synbiotic supplementation resulted in significant reduction in hs-CRP (93).	Patients with liver metastases had higher CRP (36).	Lower CRP level may indicate better outcomes, and certain treatment found to decrease level.
IDO-1	Decreased level of IDO-1 may indicate improved disease outcome (87).	Lower level of IDO-1 links the marker to possible detection of reduced disease progression in pancreatic cancer patients (94).	Lower level of IDO-1 indicate better disease outcome and can track disease status.
IL-1β	No differences in IL-1β level after regional anesthesia of radical mastectomy (69).	No significant differences (95).	No change or indication from current studies.
IL-2	IL-2 was significantly lower with treatment (73).	Patient with high IL-2 levels in their sera showed a worse overall survival (24).	Lower level of IL-2 may indicate better outcome and treatment can lower marker.
IL-6	IL-6 decreased with treatment (84). IL-6 decrease with treatment (73). IL-6 reduced (77). After RT-HIlT, IL-6 is less pronounced, reduced chemotherapy induced inflammation (89). No significant changes (88). Not a major marker in mediating sleep outcomes (52). No differences (69). No clear association (70).	IL-6 decreased with treatment (38). Decreased IL-6 (94). Higher IL-6 related to poorer overall survival (39). IL-6 negatively and significantly associated with disease control rate (41). No differences (40).	Lower IL-6 indicates better prognosis (overall survival) for the majority of trials reviewed.
IL-8	Patients with lower baseline plasma levels of IL-8 were more likely to respond to treatment and showed a longer overall survival (91). Level of IL-8 was significantly lower after San Huang decoction treatment (73). Lower plasma IL-8 during chemotherapy can predict excellent long-term survival (81).	Long-term low levels of plasma IL-8 during chemo-immunotherapy may be prognostic markers of clinical outcomes because elevated levels of circulating IL-8 are associated with poorer outcome (37). Lower IL-8 was significantly associated with overall survival (38). Increases in IL-8 was significantly associated with higher hazard of progression (41). High IL-8 showed poor survival (36). Mice bearing shTAK1 tumors had significantly lower plasma levels of IL-8 and experienced a significant reduction in tumor growth if treated with nal-IRI (44).	Lower level of IL-8 indicate better survival.
IL-6/IL-8 ratio	IL6/IL8 ratio larger than 2.0 induced cellular proliferation of MCF-7 (breast cancer transformed cell line) - more cancer cell proliferation, worse disease prognosis (60).	Chemo-immunotherapy decreased IL6/IL8 ratio, which improves disease outcomes (37).	High IL6/IL8 ratio may indicate worse cancer outcome.
IL-10	Intervention group (anti-inflammatory dietary intervention) participants had higher level of IL-10 (63).	Patients with detectable IL-10 had a better survival rate compared with patients without IL-10 in the serum (42). IL-10 was upregulated with treatment FOLFIRINOX (24).	Higher IL-10 levels can be achieved with treatment, indicates better outcome.
Natural Killer (NK) cells	NK cells increased with treatment, which improved cellular immunity (62). Significantly increased level of NK cells in HER2 positive patients (27). NK cell activity increased significantly within the mindfulness-based stress reduction group (29).	High NK cells level was associated with patient survival (24).	High NK cell level might indicate better outcomes and is responsive to inflammation-reducing non-drug therapy.
VEGF	VEGF was increased in the drainage fluids of patients treated with sapylin (76).	VEGF increases in patients receiving pelareorep + carboplatin/paclitaxel regimen (increased level of immuno supression) (41).	VEGF increases with treatment, likely indicating poorer outcomes.

c-Reactive Protein (CRP); high specificity, (hs-); Indoleamine-pyrrole 2,3-dioxygenase, (IDO); Interleukin, (IL); tumor necrosis factor alpha, (TNF-a); and vascular endothelial growth factor, (VEGF). References are indicated in [].

Besides the studies that have demonstrated utility in measuring both IL-6 and IL-8 individually in breast cancer and pancreatic cancer, the IL6/IL8 ratio is also an important factor that is currently being used to indicate disease outcomes. Studies included in our review shown on **Table 5**, are consistent in both cancer types that having a high IL6/IL8 ratio may indicate worse cancer outcome. Considering breast cancer alone, a study analyzing the role of IL6/IL8 ratio on cell proliferation, researchers showed having an IL6/IL8 ratio larger than 2.0 will induced cellular growth in a transformed cell line (60). For pancreatic cancer, after analyzing plasma IL6/IL8 levels in patients receiving chemoimmunotherapy, improved disease outcome was showed by treatment-induced IL6/IL8 decrease (37). Because the individual IL-6 and IL-8 markers show similar clinical significance related to disease progression and survival outcomes in both cancer types, and more importantly their corresponding IL-6/IL-8 ratio does also; examples like these strengthen our premise that comparing clinically relevant inflammatory biomarkers in breast cancer can inform on potentially relevant inflammatory markers in pancreatic cancer that have yet to reach the realm of clinical utility.

Another emerging inflammatory biomarker identified in studies of both breast and pancreatic cancer was IDO-1, again related to tryptophan metabolism. The results for IDO-1 were actually consistent in both types of cancers. Although the conclusions are very limited at this point, because we only had one study for each disease type that mentioned it in this review. In the breast cancer study, investigators directly measured the level of IDO-1 (87). In contrast, for pancreatic cancer it was measured indirectly (94); but both studies demonstrated that a lower level of IDO-1 associated with better disease outcomes. Thus, at this point, IDO-1 can be a potentially effective treatment target for both cancers, but further research is clearly needed to determine the validity of these initial findings. Since breast cancer is more well-understood and more people are diagnosed with the disease compared to pancreatic cancer, more research previously performed on breast cancer models and in study trials with patient participants that have identified and validated biomarkers, can be a wealthy resource from which to further explore relevance and effectiveness in pancreatic cancer.

Chemokine ligand 2 (CCL2) is a chemokine produced by osteoblasts and has important roles in cancer pathogenesis. Previous research has shown that an upregulation of the ratio of CCL2 to it receptor (CCL2/CCR2) in another hormonally-responsive cancer - prostate cancer - was associated with advancement of disease, metastasis and relapse (117). In breast cancer, a specific increase of CCL2 alone is also associated with cancer metastasis promotion; and when CCL2 was found overexpressed in breast cancer tissues, those patients had a poorer prognosis (84). These and similar findings led researchers to investigate whether CCL2 inhibition could be a feasible treatment target, and resulting tests with propagermanium (PG, a CCR2 inhibitor) (118, 119) did in fact demonstrate great effectiveness for preventing metastasis, translating to improved breast cancer outcomes (84). Using a comparable strategy to improve pancreatic cancer outcomes was not as clear-cut, when Noel and colleagues used a different type of small molecule antagonist inhibitor of CCR2, under a phase 1b study of combined treatment with nab-paclitaxel/gemcitabine in metastatic pancreatic cancer patients. Here, while they achieved a decrease of CD14+CCR2+ inflammatory monocytes in the peripheral blood samples, only 2 of 21 patients had a decrease CCR2+ tumor associated macrophages (45). Unfortunately, this response was not considered as significant efficacy compared to only using nab-paclitaxel and gemcitabine (45). Additional studies that use other therapeutics to inhibit CCL2 either alone or indirectly by targeting the CCR2 to diminish the ratio, as with the alternative medicinal inhibitory organometallic compound PG, might be needed to confirm whether CCL2 is an important contributor to disease indication or outcome in pancreatic cancer. We suggest the most obvious path should begin with trying drugs that have already shown efficacy in breast cancer trials.

Leptin is “a mediator of adipose tissue endocrine functions, such as appetite control and energy homeostasis. Leptin signaling is involved in several physiological processes as modulation of innate and adaptive immune responses and regulation of sex hormone levels (120, 121). Moreover, leptin is important in both initiating and fostering disease progression through its links to an obesogenic environment which allow deleterious crosstalk between three other mechanisms in addition to adipose-derived chronic inflammation, including the interplay with the leptin receptor, dysfunction in endocrine regulation of the sex hormones, as well as the altered signaling networks triggered by elevated levels of circulating insulin and IGF-1 receptors (122). High leptin production caused by the dysfunction of or excess adipose tissue is often seen in obesity and cancer, notably in breast cancer (123). Based on the inclusion criteria for this review, two papers identified leptin measurement as a useful breast cancer inflammatory biomarker, particularly in relation to exercise (88) or loss of body fat (64), and the potential for these two factors to decrease of leptin levels, resulting in improved the disease outcome. In the study that analyzed the effect of exercise on localized breast cancer, although leptin was decreased in both the intervention group and control group (i.e., with no significant difference in biomarker levels between groups), authors pointed out that high leptin level and low adiponectin level were also associated with increased risk of breast cancer recurrence and mortality (88). Thus, besides leptin alone, the adiponectin/leptin ratio is also associated with indication of breast cancer progression (123). A multiethnic case-control study further demonstrated that a reduced adiponectin/leptin ratio was associated with increased risk of postmenopausal breast cancer, which was also shown in preclinical models (124). Regarding the other study for this review, which supports these findings, researchers also made extra note of the relationship between leptin and body fat distribution, as a mediator of exercise intervention, and clearly demonstrated that the loss of intra-abdominal fat was associated primarily with the decrease in leptin level (64). Due to the characteristic of adiponectin/leptin ratio in obesity in general, there is strong possibility that the ratio would also serve as an excellent marker for pancreatic cancer, similar to IL-6/IL-8 ratio, for tracking inflammation for clinical purposes. Future studies should focus on whether leptin alone or the adiponectin/leptin ratio is diagnostic or useful for treatment or survival indications for pancreatic adenocarcinoma patients, and especially ones who also have the co-morbid condition of obesity.

**Figure 2:**
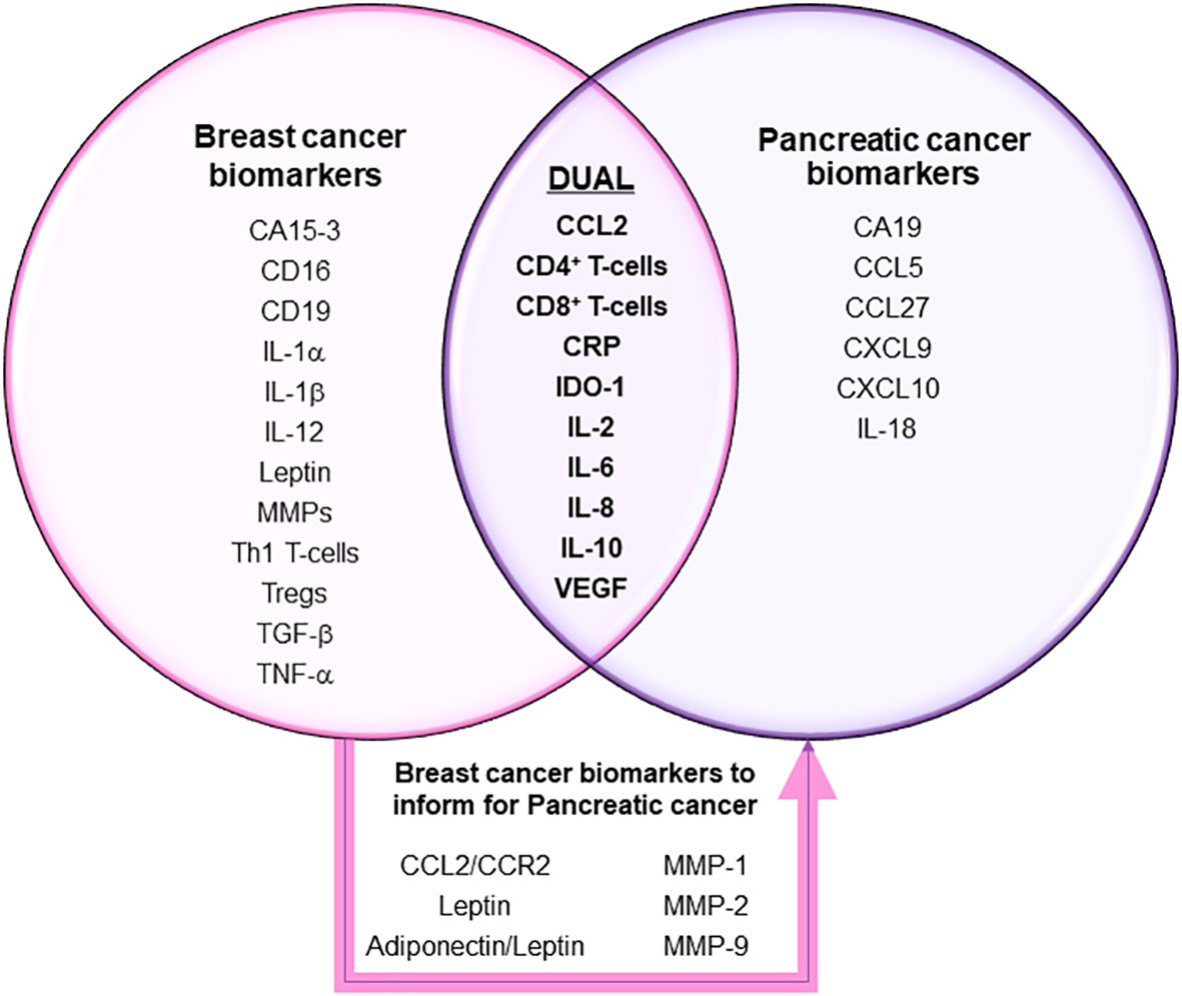
Clinically relevant inflammatory biomarkers in breast and pancreatic cancers. Markers in each sphere of the Venn diagram have been validated in many clinical trials to show clinical utility for diagnosis, prognosis and/or disease outcome indications for treatment or survival in breast and/or pancreatic cancers. Additional breast cancer-specific markers highlighted within the box represent ones that may also show usefulness for pancreatic cancer.

Matrix metalloproteinases (MMPs) are a class of zinc-dependent proteins that play important roles in the breakdown of extracellular matrix and participate in various physiological and pathological processes such as inflammation and cancer metastasis (125). In the two studies that measured and found indications for MMPs breast cancer outcomes; the first explored the effect of a novel non-steroidal anti-inflammatory drug (NSAID), β-D-Mannuronic acid (M2000), on gene expression in breast cancer patients, where researchers found that NSAID use reduced the levels of MMP-2 and MMP-9 gene expression (75). Both proteins play a role in promoting tumorigenesis and metastasis, thus the treatment and resulting reduction in biomarker levels indicated improvement of disease outcome. Other studies beyond those selected for review also support this finding, where the overexpression of MMP2- and MMP-9 has been associated previously with advanced clinical stage and histological grade (125). In addition to MMP-2 and MMP-9, MMP-1 has also been used as an accurate marker in a three markers scores model, including MMP-1, hepatocyte growth factor, and chemokine ligand 5, to predict axillary lymph node metastasis (LNM) positivity in breast cancer patients (65). Although it was independently associated with lymph node metastatic spread, only the LNM score (LNMS) based on the combination of the markers significantly predicted whether a patient would be considered as having low risk for positive nodes (LNMS=0) up to having high risk for positive nodes (LNMS≥2). Regarding utility in pancreatic cancer, most recent evidence analyzing a panel of collagen-cleaving MMPs showed that high mRNA expression of the MMPs is associated with poor survival (126). Based on its characteristic function of matrix breakdown and established evidence in breast and other cancers to contribute to metastatic spread, MMPs will likely soon emerge as clinically useful markers in pancreatic cancer; where they may be of specific use in earlier detection, as most pancreatic cancer is detected in later stages or when metastasis has already occurred. **Figure 2** represents a quick reference to cancer-specific, over-lapping cancer inflammatory biomarkers, as well as those that might be useful in pancreatic cancer.

### 4.2 Conclusion

In conclusion, breast cancer and pancreatic cancer are two distinctive cancer types with several common characteristics in aspects of gene, hormone, and inflammatory biomarkers such as IL-6, CD4 or CD8 cells, VEGF and IDO-1. Overall, the similarity in how both types of cancers respond to or result in further disruptive inflammatory signaling, which points to a list of biomarkers that have been shown useful in diagnosis, prognosis and treatment or survival outcomes strengthens the similarity between them. Further, it supports a strategy of investigating markers to date, only found clinically useful in breast cancer, to provide clues for expanding the knowledge-base for developing the same or more useful diagnostic and treatment measurement inflammatory biomarkers in pancreatic cancer. More research is needed in exploring the common ground for both breast cancer and pancreatic cancer, and this review helps serve as a roadmap to conceivably do the same in other endocrine-regulated cancers.

## Author contributions

JP and DS conceived of the review topic. JP and DS outlined the content for the review. JP performed the systematic literature search. SM, AC and DS performed fact checking for the included references. JP drafted the manuscript. JP and DS revised the manuscript, all authors critically reviewed, made final revisions and approved the submitted version of the manuscript.

## Supplementary material

Supplementary Table 1 Summary of all studies included in review, with details for study design, demographics and conclusions. NA (not applicable), wk (week/weeks), PDAC (pancreatic ductal adenocarcinoma) and ELISA (enzyme-linked immunosorbent assay).
