# Supplementary material for: Clinical trial-identified inflammatory biomarkers in breast and pancreatic cancers
Source: Front Endocrinol (Lausanne). 2023 Apr 27;14:1106520. doi: 10.3389/fendo.2023.1106520 (PMC10173309; doi:10.3389/fendo.2023.1106520)
Supplement: Supplementary Table 1 — Summary of all studies included in review, with details for study design, demographics and conclusions. NA (not applicable), wk (week/weeks), PDAC (pancreatic ductal adenocarcinoma) and ELISA (enzyme-linked immunosorbent assay). [file Table_1.docx]

**Supplementary Table 1. Summary of all studies included in review, with details for study design, demographics and conclusions.**

| **Name of Study** | **Study Type** | **Cancer Type** | **Gender** | **Age Range** | **Pre- vs Post- menopause** | **Race/ Ethnicity** | **Study Design** | **Study Conclusion** | **Biomarkers** | **Ref** |
| --- | --- | --- | --- | --- | --- | --- | --- | --- | --- | --- |
| Prognostic and predictive value of immunological parameters for chemoradioimmunoth-erapy in patients with pancreatic adenocarcinoma | clinical trial | pancreatic cancer | M/F | 33-77 | NA | NA |  | Immunological parameters, identified in this trial as possible markers, may be of interest in personalized medicine towards the improvement of the treatment and prognosis of pancreatic carcinoma patients. | Diagnostic: CD4, NK, Neutrophils, Eff CD8, IL-10, MUC, CRIt only: CD21+ mono-cytes, CCR7+, CD21+ lymphocytes, Naïve CD8, eff-mem CD8, CD152+CD4, IL-2, neutrophils, MUC, CD3, CA19 (GrB) CRt only: cytotoxicity, MUC, CA19 (GrB), Eff CD8 | (24) |
| BL-8040, a CXCR4 antagonist, in combination with pembrolizumab and chemotherapy for pancreatic cancer: the COMBAT trial | phase I, open-label, two -cohort study | pancreatic cancer | M/F | 46-86 | NA | Israel, USA, Korea | In cohort 1, 37 patients with chemotherapy-resistant disease received BL-8040 and pembrolizumab; In cohort 2, 22 patients received BL-8040 and pembrolizumab with chemotherapy, with an ORR, DCR and median duration of response of 32%, 77% and 7.8 months, respectively | combined CXCR4 and PD-1 blockade may expand the benefit of chemotherapy in PDAC and warrants confirmation in subsequent randomized trials. | CD4+ CD69+ T cells, CXCR4-expressing CD4+ and CD8+ T cells; Treg cells (CD4+ CD25+ FoxP3+) | (25) |
| The tumor-targeting immunocytokine F16-IL2 in combination with doxorubicin: dose escalation in patients with advanced solid tumors and expansion into patients with metastatic breast cancer | open-label, non-randomized, phase Ib/II study | breast cancer | M/F | 32-77 | NA | NA | Nineteen patients with progressive solid tumors were enrolled in the phase Ib part of the study (Table 1) and treated with increasing doses of F16- IL2 (Fig. 2A). | F16-IL2 can be safely and repeatedly administered at the RD of 25 MIU in combination with 25 mg/m2 doxorubicin; its safety and activity are currently being investigated in combination with other chemotherapeutics, in order to establish optimal therapy settings. | immunogenic response not showed | (26) |
| Improved Natural Killer cell activity and retained anti-tumor CD8(+) T cell responses contribute to the induction of a pathological complete response in HER2-positive breast cancer patients undergoing neoadjuvant chemotherapy | phase II mono-institutional trial | breast cancer | F | 23-70 | NA | NA | The immune profile of 40 HER2-positive and 38 HER2-negative BC patients was characterized at diagnosis and throughout NC (Paclitaxel and Trastuzumab, or Docetaxel and Epirubicin, respectively). The percentages of circulating immune cell subsets including T and B lymphocytes, Natural Killer (NK) cells, regulatory T cells, T helper 17 lymphocytes, were quantified by multiparametric flow cytometry. NK cells functional activity was evaluated through the analysis of NF-kB nuclear translocation by Multispectral flow cytometry, and with the in vitro monitoring of Trastuzumab-mediated antibody-dependent cell cytotoxicity (ADCC). CD8+ T cell responses against six different tumor-associated antigens (TAA) were characterized by IFN-γ ELISPOT and IFN-γ/IL-2 DualSpot assays. | These results indicate that maintenance of functional T cell responses against selected antigens and improvement of NK cell proficiency during NC are probably critical requirements for pCR induction, especially in HER2-positive BC patients. | NK cells, regulatory T cells; T helper 17 cells; CD8+ T cell, CD4+ T cell | (27) |
| High-circulating Tie2 Is Associated With Pathologic Complete Response to Chemotherapy and Antiangiogenic Therapy in Breast Cancer | prospective phase II study | breast cancer | F | no range | NA | African American, Caucasian | We have conducted a prospective phase II study in 39 patients using bevacizumab in breast cancer in the neoadjuvant setting and found improved pathologic complete response (pCR) when bevacizumab was added to chemotherapy in patients with hormone receptor negative and invasive ductal carcinoma. | Baseline circulating Tie-2 levels may help distinguish patients who will have pCR from those who will not and may form the basis for future development of antiangiogenic therapy in breast cancer. Larger studies are needed to validate these findings. | Tie-2, bFGF | (28) |
| Mindfulness and its efficacy for psychological and biological responses in women with breast cancer | randomized controlled trial | breast cancer | F | 34-80 | NA | NA | This RTC assigned 166 women with breast cancer to one of three groups: MBSR (8 weekly group sessions of MBSR), active controls (self-instructing MBSR) and non-MBSR. The primary outcome measure was the Hospital Anxiety and Depression Scale. Secondary outcome measures were: Memorial Symptom Assessment Scale, SF-36, Sense of Coherence, Five Facets of Mindfulness Questionnaire, and Post-traumatic Growth Index. Blood samples were analyzed using flow cytometry for NK-cell activity (FANKIA) and lymphocyte phenotyping; concentrations of cytokines were determined in sera using commercial high sensitivity IL-6 and IL-8 ELISA kits. | In conclusion, results from this RCT suggest that MBSR is beneficial and leads to psychological and biological improvements. MBSR may hold potential for alleviating depression, distress and symptom experience, and to strengthen coping capacity, which may improve breast cancer survivorship. | NK cells (CD3-16+56+); CD3+ & CD3+8+ T-lymphocytes; CD19+ B-lymphocytes, CD19B% | (29) |
| Binding of circulating anti-MUC1 antibody and serum MUC1 antigen in stage IV breast cancer | clinical trial | breast cancer | NA | 31-67 | NA | NA | Serum samples of 61 patients with stage IV breast cancer and 64 patients with early-stage breast cancer were collected. The anti-MUC1 antibody (IgG) and MUC1 antigen (cancer antigen 15-3; Ca15-3) were detected using an indirect enzyme-linked immunosorbent assay (I-ELISA) and ELISA, respectively. The MUC1 IgG affinity was detected using a urea degradation combining ELISA. Western blot analysis and an inhibition test were performed for verification of the binding of anti-MUC1 IgG with MUC1 antigen, and their correlation was analyzed | In conclusion, circulating anti-MUC1 antibody was found to bind serum MUC1 antigen in stage IV breast cancer, however, the compatibility of anti-MUC1 antibody and MUC1 antigen may be low. No significant difference was found in the affinity of anti-MUC1 antibody between stage IV breast cancer and early-stage breast cancer. | IgG, CA 15-3 | (30) |
| Receptor activator of nuclear factor kappa B (RANK) expression in primary breast cancer correlates with recurrence-free survival and development of bone metastases in I-SPY1 (CALGB 150007/150012; ACRIN 6657) | multicenter study | breast cancer | F | no range | Both | NA | We evaluated RANK/RANKL/OPG expression using expression microarrays in I-SPY1 (n=149). Associations with clinical features were determined using t-test and ANOVA. Associations between biomarker High vs Low groups (dichotomized at an optimal cut point) and recurrence free survival (RFS) were evaluated using the log rank test and in a multivariate Cox proportional hazard model. A pooled external neoadjuvant cohort with gene expression data (GSE25066) [1] (n=425) was used for validation. Associations with site-specific relapse were evaluated using the t-test and multivariate logistic regression adjusting for hormone receptor (HR) status. | RANK is increased in HR negative and basal BC, and correlates with worse RFS and risk of BM. The RANK pathway is a potential therapeutic target in BC. | Receptor Activator of Nuclear Factor Kappa B | (31) |
| Dynamic changes of Receptor activator of nuclear factor-κB expression in Circulating Tumor Cells during Denosumab predict treatment effectiveness in Metastatic Breast Cancer | pilot study | breast cancer | F | 32-89 | both | NA | All the patients received the recommended dose of 120 mg denosumab, as a single subcutaneous injection once every 4 weeks - into the thigh, abdomen or upper arm, for prevention of skeletal related events in adults with bone metastases from solid tumors | In conclusion, we demonstrated, for the first time, the presence of RANK-positive CTCs in bloodstream of MBC patients and that in vivo the effectiveness of Denosumab depends on the expression of RANK. Our findings offer a rationale to design future prospective clinical trials for changing vs continuing initial therapy, utilizing the “brief ” CTC test for RANK as predictive marker. More in general, we provided evidence that CTCs allow monitoring serial changes of cancer biology, revealing the spatial and temporal heterogeneity of individual tumor undergone a specific treatment. | RANK in CTCs | (32) |
| Effects of Molecular Iodine/Chemotherapy in the Immune Component of Breast Cancer Tumoral Microenvironment | clinical trial | breast cancer | NA | NA | NA | NA | Thirty patients were randomly assigned (double-blind) to receive either molecular iodine (I2; 5mg/day) or a placebo (vegetable colored water) for 7–35 days (as determined by the preoperative oncologist’s protocol). In the Advanced group, 30 patients were randomly (double-blind) divided into the I2 or placebo groups, and both groups received 4–6 cycles of neoadjuvant chemotherapy (Cht; 5-fluorouracil /epirubicin/cyclophosphamide or taxotere/epirubicin). | In conclusion, our data showed that I2 supplements induce the activation of the immune response and that when combined with Cht, the Th1 pathways are stimulated. The molecular mechanisms involved in these responses are being analyzed, but preliminary data suggest that methylation/demethylation mechanisms could also participate. | TH1, TH17, M0 macrophages, B lymphocytes | (33) |
| Efficacy and Determinants of Response to HER Kinase Inhibition in HER2-Mutant Metastatic Breast Cancer | clinical trial | breast cancer | M/F | 37-87 | both (but majority post-menopausal) | NA | In total, 81 patients with HER2-mutant metastatic breast cancer were enrolled, including 34 patients who received neratinib monotherapy (23 HR+, 11 HR negative [HR–]) and 47 who received neratinib plus fulvestrant (all HR+). | Collectively these data define HER2 mutations as a therapeutic target in breast cancer and suggest that co-existence of additional HER signaling alterations may promote both de novo and acquired resistance to neratinib. | HER2, HER3 | (34) |
| Intratumoral Hydrogen Peroxide With Radiation Therapy in Locally Advanced Breast Cancer: Results From a Phase 1 Clinical Trial | clinical trial | breast cancer | M/F | 45-93 | NA | NA | Thirteen patients (11 female, 2 male) were recruited to the study between February 2017 and August 2018. All patients had locally advanced or recurrent breast cancer and were inoperable due to comorbidities, local extent of disease, or metastatic disease. | Intratumoral H2O2 with RT is well tolerated with no additional toxicity compared with RT alone. If efficacy is confirmed in a randomized phase 2 trial, the approach has potential as a cost-effective radiation response enhancer in multiple cancer types in which locoregional control after RT alone remains poor | IL-1b, IL-4, MTP-1a | (35) |
| Phase I pilot study of Wilms tumor gene 1 peptide-pulsed dendritic cell vaccination combined with gemcitabine in pancreatic cancer | phase I pilot study | pancreatic cancer | M/F | 41-69 | NA | NA | The remaining 10 patients (4 with locally advanced and 6 with metastatic PC) had a median age of 58 years (range, 41–69 years). Five patients (50%) completed the protocol, while 5 (50%)terminated the protocol because of rapid disease progression or a severe adverse event; | In summary, the present study found that DCGEM therapy was feasible, tolerable and effective as a first-line therapy for inducing anti-tumor T-cell responses in patients with advanced PC without liver metastases. | tetramer-positive WT 1-specific T cell; NLR, CRP, IL-8 | (36) |
| Prognostic significance of plasma interleukin-6/-8 in pancreatic cancer patients receiving chemoimmunotherapy | clinical trial | pancreatic cancer | M/F | 35-72 | NA | NA | All 7 patients with PDA received gemcitabine followed by a combination of gemcitabine and biweekly vaccinations with DC/WT1-Ⅰ/Ⅱ (Figure 1). The clinical characteristics of all PDA patients are presented in Table 1. All patients had disease stage Ⅳ and HLA types of A (A*02:01, A*02:06, or A*24:02), DR (DRB1*04:05, DRB1*08:03, DRB1*15:01, or DRB1*15:02) or DP (DPB1*05:01, or DPB1*09:01). | Prolonged low levels of plasma IL-6/-8 in PDA patients may be a prognostic marker for the clinical outcomes of chemoimmunotherapy. | IL-6, IL-8 | (37) |
| Phase II trial of salvage therapy with trabectedin in metastatic pancreatic adenocarcinoma | single-center, prospective, single-arm phase II study | pancreatic cancer | M/F | 48.3-73.8 | NA | NA | 25 patients with metastatic pancreatic adenocarcinoma were enrolled in the trial. | Although it has shown some ability to modulate inflammatory process, single-agent trabectedin had no activity as salvage therapy for mPA. | CCL27, CXCL9, CXCL 10, IL-6, and TRAIL; PTX3 and MIF; IL-6, IL-8, CXCL1, IL-Ra, CXCL12, IFNa2, PTX3, HGF, and SCGF | (38) |
| Systemic immune activity predicts overall survival in treatment naive patients with metastatic pancreatic cancer | phase II clinical trials | pancreatic cancer | M/F | 39-84 | NA | American Indian, Asian, African American, Caucasian, Other | Peripheral blood was obtained from 73 patients with histologically confirmed, inoperable/metastatic PDAC who were treatment naïve | These data support the hypothesis that baseline immune status predicts PDAC disease course and overall patient survival. | higher IL-6, IL-10, CTLA-4 on CD8+ T cell; higher MCP-1,CD45RO+, TIM3 on CD4+ cell | (39) |
| Immunobiological effects of gemcitabine and capecitabine combination chemotherapy in advanced pancreatic ductal adenocarcinoma | clinical trials | pancreatic cancer | M/F | 44-79 | NA | NA | The levels of granulocyte macrophage-colony-stimulating factor (GM-CSF) and interleukin-6 (IL-6) and the associated inflammatory marker C-reactive protein (CRP) were assessed in 38 patients receiving gemcitabine and capecitabine combination chemotherapy for advanced pancreatic cancer within the TeloVac trial | Combination gemcitabine and capecitabine chemotherapy did not affect circulating levels of GM-CSF, IL-6 and CRP. Chemotherapy-induced apoptosis was not associated with the immunogenicity induced by the GV1001 vaccine in advanced pancreatic cancer. | CRP, IL-6, GM-CSF | (40) |
| Randomized Phase 2 Trial of the Oncolytic Virus Pelareorep (Reolysin) in Upfront Treatment of Metastatic Pancreatic Adenocarcinoma | randomized phase 2 trial | pancreatic cancer | M/F | 39-84 | NA | American Indian, AS, African American, Caucasian, Other | Seventy-six patients were randomized between February 2011 and April 2014. Three patients withdrew from the study prior to receiving any therapy and were not considered evaluable, resulting in 73 patients evaluable for the primary endpoint of PFS: 36 on Arm A and 37 on Arm B | Overall, pelareorep was safe but does not improve PFS when administered with carboplatin/paclitaxel, regardless of KRAS mutational status. Immunologic studies suggest that chemotherapy backbone improves immune reconstitution and that targeting remaining immunosuppressive mediators may improve oncolytic virotherapy | IL-6, IL-8, VEGF, regulatory T cell, CTLA4 on both CD+ and CD8+ | (41) |
| Phase 1b study targeting tumour associated macrophages with CCR2 inhibition plus FOLFIRINOX in locally advanced and borderline resectable pancreatic cancer | single-center, open label, phase Ib clinical trial | pancreatic cancer | M/F | 41-75 | NA | Caucasian, Black, other | From April 19th, 2012 through November 12th, 2014 a total of 47 patients were enrolled. The dose de-escalation group (n=6) received PF-04136309 at 500 mg administered orally twice daily. No dose-limiting toxicities were observed and this was established as the recommended phase 2 dose. The expansion phase cohort (n=33) and patients in the dose de-escalation arm receiving PF-04136309 at the recommended phase 2 dose (n=6) were combined for assessment of treatment related toxicity | CCR2 targeted therapy with PF-04136309 in combination with FOLFIRINOX is safe and tolerable. Corollary studies suggest that CCR2 blockade reduces TAM and alters the TME, providing rationale for future clinical studies of this promising treatment modality | inflammatory monocyte (CCR2+); IL-12, TNFa, CD8+ TIL, Helper CD4+; IL-4, IL-10, IL-13, TGF-b; FoxP3 regulatory T-cell | (42) |
| Pharmacogenomic analyses of sunitinib in patients with pancreatic neuroendocrine tumors | phase IV trial | pancreatic cancer | M/F | no range | NA | White, Black, Asian | From a total of 106 patients enrolled in the clinical study, 56 patients (25 treatment-naive and 31 previously treated) consented, provided a blood sample and were genotyped. | IL1B SNPs may predict treatment response in patients with pancreatic neuroendocrine tumors. VEGF pathway SNPs are potentially associated with survival outcomes. | IL1B | (8) |
| Phase I clinical trial repurposing all-trans retinoic acid as a stromal targeting agent for pancreatic cancer | phase 1b randomized control trial | pancreatic cancer | M/F | 27-78 | NA | White, Asian, Black, Other | We used an innovative two-step, adaptive, Bayesian continual reassessment method using five potential dose levels (DL) which appears to have advantages over standard 3 + 3 and titeCRM designs in accurately predicting RP2D, based on priors of toxicity data13 (Fig. 1, Supplementary Fig. 1, and Supplementary Table 1). A total of 32 patients were screened to enroll 28 of whom 27 received any treatment from February 2016 to February 2018.  clinical parameters was 1 April 2019. | Baseline stromal-specific retinoid transport protein (FABP5, CRABP2) expression may be predictive of response. | FABP5, CRABP2, PTX3 | (43) |
| Plasma IL8 Is a Biomarker for TAK1 Activation and Predicts Resistance to Nanoliposomal Irinotecan in Patients with Gemcitabine-Refractory Pancreatic Cancer |  | pancreatic Cancer | M/F | 32-81 | NA | NA | Patients enrolled in this study had advanced histologically or cytologically confirmed pancreatic adenocarcinoma and were previously treated with gemcitabine-based therapy, received in localized or metastatic setting | Our study identified IL8 as the most significant circulating factor for TAK1 pathway activation and candidates IL8 as a potential predictive biomarker of resistance to nal-IRI in gemcitabine-refractory patients with pancreatic cancer. | IL8 | (44) |
| Phase 1b study of a small molecule antagonist of human chemokine (C-C motif) receptor 2 (PF-04136309) in combination with nab-paclitaxel/ gemcitabine in first-line treatment of metastatic pancreatic ductal adenocarcinoma | phase 1b study | pancreatic cancer | M/F | 46-79 | NA | White, Black, Asian | Eligible patients were males and females ≥18 years of age, with histologically or cytologically proven diagnosis of mPDAC who had provided a baseline tumor sample at registration. Patients had not received previous radiotherapy, surgery, chemotherapy, or investigational therapy for the treatment of metastatic disease and had a life expectancy ≥12 weeks. Patients with Eastern Cooperative Oncology Group performance status (ECOG PS) 0 or 1 and adequate bone marrow, renal, and liver function were included. Patients with known symptomatic brain metastases requiring steroids or who had prior therapy with modulators of monocyte or TAM function in metastatic setting were ineligible to participate. | PF-04136309 in combination with nab-paclitaxel plus gemcitabine had a safety profile that raises concern for synergistic pulmonary toxicity and did not show an efficacy signal above nab-paclitaxel and gemcitabine. | CCL2, CD14 + CCR2+ inflammatory monocytes (IM) | (45) |
| A phase 1b trial of concurrent immunotherapy and irreversible electroporation in the treatment of locally advanced pancreatic adenocarcinoma | phase 1b clinical trial | pancreatic cancer | M/F | 38-67 | NA | Non-Hispanic white, Hispanic | This was a clinical trial (clinical trial #NCT03080974) approved by our institutional review board (IRB) and a data safety monitored board (DSMB) and carried out at the Louisville Medical Center. Key inclusion criteria were age 18 years; glomerular filtration rate > 30 mL/min/1.73 m2; aspartate aminotransferase and alanine aminotransferase levels within 3 times the upper limit of normal; measurable disease on biaxial dimensional analysis, such as triple phase computed tomography (CT) or intraoperative ultrasonography; and stage III, histologically confirmed, unresectable locally advanced PDAC defined as greater than 180  encasement of the major arterial structures (ie, superior mesenteric artery or celiac axis) without evidence of metastatic disease including the liver or peritoneum | Irreversible electroporation induces expression of PD-L1 in vitro. Combination therapy with concurrent nivolumab is well tolerated. A multicenter, phase 2 adjuvant trial is underway using irreversible electroporation and nivolumab in patients with locally advanced pancreatic cancer. | T-effector memory cell and PD-L1 | (46) |
| Randomized Phase III Study of FOLFOX Alone or With Pegilodecakin as Second-Line Therapy in Patients With Metastatic Pancreatic Cancer That Progressed After Gemcitabine (SEQUOIA) | randomized phase III study | pancreatic cancer | M/F | 33-88 | NA | Asian, Europe, North America | SEQUOIA, a randomized, global phase III study, compared FOLFOX with PEG 1 FOLFOX as second line in gemcitabine-refractory PDAC. Patients were randomly assigned 1:1 (PEG1 FOLFOX: FOLFOX) and stratified by prior gemcitabine and region. Eligible patients had only one prior gemcitabine containing treatment. Primary end point was overall survival (OS). Secondary end points included progression free survival (PFS), response evaluation per Response Evaluation Criteria in Solid Tumor (RECIST) 1.1, and safety. Exploratory analyses included biomarkers related to immune activation. | PEG added to FOLFOX did not improve efficacy in advanced gemcitabine-refractory PDAC. Safety findings were consistent as previously observed from PEG with chemotherapy; toxicity was manageable and tolerable. Exploratory pharmacodynamic results were consistent with immunostimulatory signals of the IL-10R pathway. | IL-18, interferon-y, granzyme B; TGF-beta | (47) |
| Immunologic and tumor responses of pegilodecakin with 5-FU/LV and oxaliplatin (FOLFOX) in pancreatic ductal adenocarcinoma (PDAC) | multi-institutional, open-label, multiple-cohort, dose-escalation, phase 1b study. | pancreatic cancer | M/F | 41-70 | NA | Asian, African American, White | Cohort C enrolled 39 patients (29 PDAC patients, 6 colorectal cancer patients, 2 gastric, and 2 “other” (1 neuroendocrine carcinoma of the colon and 1 liver adenocarcinoma). Of the 29 PDAC patients, 2 were provided 2.5 μg/kg, 2 received 10 μg/kg, and 25 patients received 5 μg/kg pegilodecakin. | Pegilodecakin+FOLFOX had an acceptable tolerability profile in PDAC, with no substantial irAEs seen, and promising efficacy with the combination yielding a 2-year OS of 24% (95% CI 10–42). These data led to the phase 3 study with pegilodecakin+FOLFOX as second-line therapy of PDAC (SEQUOIA). | CA19-9 | (48) |
| Circulating biomarkers and outcomes from a randomized phase 2 trial of gemcitabine versus capecitabine-based chemoradiotherapy for pancreatic cancer | randomized phase 2 trial | pancreatic cancer | M/F | no range | NA | NA | Briefly, patients with histologically/cytologically confirmed inoperable locally advanced pancreatic cancer with maximum diameter 7 cm or less, performance status (PS) 0–2, were eligible. | CCL5 is an independent prognostic biomarker in LAPC. Given the known role of CCL5 in tumour invasion, metastasis and the induction of an immuno-suppressive micro-environment, targeting of CCL5-mediated pathways may offer therapeutic potential in pancreatic cancer. | CCL 5 | (49) |
| Effects of Fresh Yellow Onion Consumption on CEA, CA125 and Hepatic Enzymes in Breast Cancer Patients: A Double-Blind Randomized Controlled Clinical Trial | double-blind randomized controlled clinical trial | breast cancer |  | 30-63 | NA | NA | BC patients whose disease had been approved histopathologically after radical or partial mastectomy in Nour-Nejat hospital and who referred to Shahid Ghazi Cancer Research Centre and private cancer clinics (Tabriz, Iran) aged 30 to 65 years old were enrolled of the primary population of women afflicted with BC (whole date range for patient recruitment was between October 2012 till June 2013 | Our findings for the first time showed that regular onion administration could be effective for hepatic enzyme conveying adjuvant chemotherapy relevant toxicity and reducing the tumor markers in BC during doxorubicin-based chemotherapy. | ALT, AST; CA125, CEA; ALP | (50) |
| Targeted T-cell Therapy in Stage IV Breast Cancer: A Phase I Clinical Trial | phase I clinical trial | breast cancer | F | 31-68 | NA | NA | ATC were expanded from leukapheresis product using IL-2 and anti-CD3 monoclonal antibody and armed with HER2Bi. In 3+3 dose escalation design, groups of 3 patients received 5, 10, 20, or 40   109 armed ATC (aATC) per infusion. | Targeting HER2 positive and negative tumors with aATC infusions induced anti-tumor responses, increases in Th1 cytokines and IL-12 serum levels that suggest that aATC infusions vaccinated patients against their own tumors. These results provide a strong rationale for conducting phase II trials. | Th1, IL-12 | (51) |
| Inflammation and psychosocial factors mediate exercise effects on sleep quality in breast cancer survivors: pilot randomized controlled trial | pilot randomized controlled trial | breast cancer | F | 32-69 (previously reported) | post | White, other | Forty-six postmenopausal BCS (≤ Stage II, off primary treatment) were randomized to a 3-month exercise intervention or control group. Intervention included 160 minutes/week of moderate intensity aerobic walking, twice weekly resistance training (resistance bands), and six discussion groups (to improve adherence) | Inflammation and psychosocial factors may mediate or enhance sleep response to our exercise intervention. Further study is warranted to confirm our results and translate our findings into more effective interventions aimed at improving sleep quality in BCS. | Il-6, IL-8, IL-10, TNF-alpha | (52) |
| ELYPSE-7: a randomized placebo-controlled phase IIa trial with CYT107 exploring the restoration of CD4+ lymphocyte count in lymphopenic metastatic breast cancer patients | randomized placebo-controlled phase I trial | breast cancer | F | 39-76 | NA | NA | A total of 20 MBC patients were randomized (Figure 1B). Baseline characteristics are detailed in Table 1. All patients were lymphopenic at inclusion with a median CD4+ lymphocyte count of 242 cells/μl | In lymphopenic MBC, CYT107 increases CD4+ and other T-cell subset counts without altering their function. A larger clinical trial to demonstrate its impact on clinical outcome is warranted. | CD4+, CD8+ | (53) |
| A Comparison of Fentanyl and Flurbiprofen Axetil on Serum VEGF-C, TNF-α, and IL-1ß Concentrations in Women Undergoing Surgery for Breast Cancer | clinical trial | breast cancer | F | no range | NA | NA | Forty-women with primary breast cancer undergoing a modified radical mastectomy were randomized to receive postoperative analgesia with flurbiprofen axetil combined with fentanyl or fentanyl alone. Venous blood was sampled before anesthesia, at the end of surgery, and at 48 hours after surgery, and the serum was analyzed. The primary endpoint was changes in the VEGF-C concentrations in serum. | In patients undergoing a mastectomy, postoperative analgesia with flurbiprofen axetil, combined with fentanyl, were associated with decreases in serum concentrations of VEGF-C, TNF-a, and IL-1ß compared with patients receiving doses of only fentanyl | VEGF-C, TNF-alpha, IL-1beta | (54) |
| Effect of Propofol and Desflurane on Immune Cell Populations in Breast Cancer Patients: A Randomized Trial | randomized trial | breast cancer | F | no range | NA | NA | The patients were randomly assigned to receive propofol (n = 20) or desflurane (n = 20) anesthesia. The total and differential white blood cell counts were determined with lymphocyte subpopulations before and 1 hr after anesthesia induction and at 24 hr postoperatively. Plasma concentrations of interleukin (IL)-2 and IL-4 were also measured | Our findings indicate that both propofol and desflurane anesthesia for breast cancer surgery induce a favorable immune response in terms of preservation of IL-2/IL-4 and CD4+/CD8+ T cell ratio in the perioperative period. With respect to leukocytes and NK cells, desflurane anesthesia is associated with less adverse immune responses than propofol anesthesia during surgery for breast cancer. | IL-2/IL-4, CD4+/CD8+; NK cell, leukocytes | (55) |
| Postsurgical Depressive Symptoms and Proinflammatory Cytokine Elevations in Women Undergoing Primary Treatment for Breast Cancer | clinical trial | breast cancer | F | Mean: 50.4, 51.19 (no range) | NA | White non-Hispanic, Hispanic, other | Women with stage 0–III BCa were recruited approximately 4–8 weeks post-surgery. Depressive symptoms were assessed using the Hamilton Rating Scale for Depression and blood samples were collected to quantify circulating levels of IL-1β, IL-6, and TNF-α by ELISA. ANCOVAs were used to test for group differences (elevated vs. low depressive symptoms) in levels of cytokines. Multiple regression analyses were used to examine relationships between continuous severity of depressive symptoms and levels of cytokines adjusting for relevant biobehavioral covariates. | Post-surgery and pre-adjuvant treatment for early stage BCa, depressive symptoms covary with elevated levels of multiple pro-inflammatory cytokines. Findings have implications for psychosocial and biological interventions concurrently focusing on depression and inflammation. | IL-1beta, TNF-alpha | (56) |
| Association of CA27.29 and Circulating Tumor Cells Before and at Different Times After Adjuvant Chemotherapy in Patients with Early-stage Breast Cancer - The SUCCESS Trial | clinical trial | breast cancer | F | 21-78 | both | NA | The SUCCESS trial compared fluorouracil, epirubicin and cyclophosphamide followed by docetaxel vs. FEC followed by docetaxel plus gemcitabine, and 2 vs. 5 years of treatment with zoledronic acid in 3,754 patients with node-positive or high-risk node negative early-stage breast cancer. | We showed that CTC and CA27.29 positivity were significantly, but only weakly associated before CHT and 5 years after CHT, while no significant association was found immediately or 2 years after CHT during the course of early-stage breast cancer. It, therefore, seems reasonable to further evaluate the prognostic value of CTCs and CA27.29 as a combined prognostic test of two potentially independent markers that might provide complementary prognostic information. | CA27.29 (mucin-1) | (57) |
| Resistance Exercise and Inflammation in Breast Cancer Patients Undergoing Adjuvant Radiation Therapy: Mediation Analysis From a Randomized, Controlled Intervention Trial | randomized controlled intervention trial | breast cancer | F | mean 57.2 (no range) | NA | NA | Breast cancer patients scheduled for adjuvant radiation therapy were randomized to 12-week progressive resistance exercise training (EX) or a relaxation control group. Interleukin-6 (IL-6) and interleukin-1 receptor antagonist (IL-1ra) were measured in serum samples collected before, at the end, and 6 weeks after radiation therapy from 103 chemotherapy-naïve participants. | This randomized, controlled trial showed a significantly increased proinflammatory cytokine level after adjuvant radiation therapy in breast cancer patients. This effect was counteracted by progressive resistance exercise training. Interleukin-6 and the IL-6/IL-1ra ratio seemed to mediate the beneficial effect of exercise on physical fatigue and pain but only to a small extent. | IL-6, Il-6/IL-1a | (58) |
| Angiogenic cytokines and their influence on circulating tumour cells in sera of patients with the primary diagnosis of breast cancer before treatment | prospective, randomized adjuvant study | breast cancer | F | NA | NA | NA | A total of 200 patients’ sera were included in this study, 100 patients being CTC positive and 100 patients being CTC negative. Matching criteria were histo-pathological grading, lymph node metastasis, hormone receptor status, TNM classification and survived breast cancer patients vs. deceased tumor associated patients. A multi cytokine/chemokine array was used to screen the sera for the angiogenic markers | Both vascular markers showed enhanced expression in the CTC negative patient collective. To continue, the collective graded G2 showed significantly enhanced sFlt1 expressions amongst patients with no CTCs. Moreover, the patient collective with no lymph node metastasis and CTC negativity indicated statistically significant increased sFlt1 values. A functional interaction of sFlt1 and PlGF was found, suggesting that their overexpression in tumour cells inhibits CTCs entering the peripheral blood. Furthermore, in regard to CTC negativity, sFlt1 and PlGF values may potentially serve as predictive markers. | sFlt1, PIGF, VEGF, VEGF-C, VEGF-D | (59) |
| Relationship of inflammatory profile of elderly patients serum and senescence-associated secretory phenotype with human breast cancer cells proliferation: Role of IL6/IL8 ratio | clinical trial | breast cancer | M/F | 60-83 | NA | NA | we evaluated if the pro-inflammatory profile within the serum obtained from elderly patients(EPS) was able to induce cellular proliferation in the breast cancer transformed cell line (MCF-7), in a similar way to the proliferation stimulated by the SASP obtained from WI-38 primary cells prematurely induced to senescence by oxidative stress (SIPS). At the same time, the participation of IL-6/IL-8 ratio was determined. | Our results showed that not all the EPS increased MCF-7 proliferation. However, there was an interesting relationship between IL-6 and IL-8 concentrations, when the IL-6 was higher than IL-8. Similar results were found with SASP from SIPS-WI-38 on the MCF-7 proliferation. Although it is known that those cytokines are fundamental factors to induce proliferation; the occurrence of other components in the cellular microenvironment is necessary to carry out this effect. | IL-6, IL-8, IL-10 | (60) |
| Testing breast cancer serum biomarkers for early detection and prognosis in pre-diagnosis samples | case-control study | breast cancer | F | 50.3-76.5 | post | NA | This nested case–control study within the UK Collaborative Trial of Ovarian Cancer Screening (UKCTOCS) used serum samples from 239 women who subsequently developed breast cancer and 239 matched cancer-free controls. Sera were screened by ELISA for 9 candidate markers. Univariate and multivariate analyses were performed to examine associations with clinicopathological features and between case controls in different time groups before diagnosis | This study using unique pre-diagnosis samples shows that CA15-3, HSP90A and PAI-1 have potential as early prognostic markers and warrant further investigation. However, none of the candidates or combinations would be useful for screening. | CA15-3; PAI-1, HSP90A | (61) |
| Tumor cryoablation in combination with natural killer cells therapy and Herceptin in patients with HER2-overexpressing recurrent breast cancer | clinical trial | breast cancer | F | 26-71 | NA | NA | 48 patients who met the enrollment criteria were assigned to three groups (n =16): cryoablation group (group I), cryoablation-NK cells therapy group (group II) and cryoablation-NK cells therapy-Herceptin group (group III). Safety and short-term effects were evaluated. | In conclusion, the three-therapy combination of tumor cryoablation, NK cells, and Herceptin yielded good outcomes in HER2-overexpressing recurrent breast cancer patients | circulating tumor cells (CTCs), carcino-embryonic antigen (CEA), CA15-3 | (62) |
| An anti-inflammatory dietary intervention to reduce breast cancer recurrence risk: Study design and baseline data | one-year, culinary-based, pilot intervention | breast cancer | F | no range (larger than 18) | NA | black or African American, US Latino, Anglo, Native American, Asian, other | A total of 153 BCSs were recruited. Overweight and obese women aged 18 or older were randomized into Intervention (IG; n = 76) and Control (CG; n = 77) groups. CG received monthly nutritional brochures from the American Institute for Cancer Research. IG attended 6 monthly workshops (lectures on AI topics and chef-prepared food demonstrations), and received monthly newsletters and telephone calls incorporating Motivational Interviewing. | As expected, positive relationships were seen between % body fat and BMI; BMI and a major pro-I cytokine (CRP); and between pro-I cytokines IL-6, IL-8 and TNF-α. Interestingly, age was negatively correlated with the anti-inflammatory cytokine IL-10, albeit weakly. | CRP, IL-6, IL-8, TNF-alpha; IL-10 | (63) |
| Association between changes in fat distribution and biomarkers for breast cancer | three-armed RCT | breast cancer | F | no range (total mean: 60.0) | post | NA | In the SHAPE-2 trial, 243 postmenopausal overweight women were included. The intervention in this trial consisted of 5-6 kg weight loss either by diet only or exercise plus diet. After 16 weeks, we measured serum sex hormones, inflammatory markers, total body fat (measured by DEXA scan) and intra and subcutaneous abdominal fat (measured by MRI). Associations between changes in different body fat depots and biomarkers were analyzed by linear regression using the study cohort irrespective of randomization to make maximal use of the distribution of changes in fat measures. | We conclude that, in our population of healthy overweight postmenopausal women, loss of fat at different body locations was associated with changes in different types of biomarkers, known to be related to risk of breast cancer | High sensitivity CRP (hsCRP), leptin | (64) |
| Criteria derived from serum markers can precisely evaluate axillary status in breast cancer patients | clinical trial | breast cancer | F | no range | NA | NA | We tested 26 factors in serum from 57 patients with resectable breast cancer by the Luminex assay. Differences between node-negative and node-positive patients were assessed. The diagnostic value of the factors was determined by further analyses and a validation test. | The LNMS derived from matrix metalloproteinase-1, hepatocyte growth factor, and chemokine ligand 5 serum levels identified the axillary lymph node status with high accuracy. Patients with higher LNMS had a greater probability of LNM. | Matrix metalloproteinase-1, hepatocyte growth factor, chemokine ligand 5 | (65) |
| The effect analysis of CYP2D6 gene polymorphism in the toremifene and tamoxifen treatment in patient with breast cancer | clinical trial | breast cancer | F | no range | NA | NA | Seventy-eight patients who received radical mastectomy and toremifene and tamoxifen treatment after operation were divided into three groups: CYP2D6*1/*1 group (13 cases), CYP2D6*1/*10 group (28cases) and CYP2D6*10/*10 group (35 cases), according to the gene polymorphism of blood serum CYP2D6. | In summary, CYP2D6 gene polymorphism relates with the effect of toremifene and tamoxifen treatment in patient with ER positive breast cancer and null allele homozygote CYP2D6*10/*10 can lead to a poor prognosis. | CA 125, CA 153, VEGF, IGF-1 | (66) |
| Increased interleukin-35 expression in tumor-infiltrating lymphocytes correlates with poor prognosis in patients with breast cancer | clinical trial | breast cancer | F | no range | both | NA | One cohort included 110 unrelated Chinese women with IDC who underwent lumpectomy or mastectomy in the Affiliated Hospital of Binzhou Medical College between January 2008 and May 2010, allowing the assessment of progression-free survival (PFS) and overall survival (OS). The second cohort included 60 patients with newly diagnosed IDC from March 2014 to December 2014 and 30 age-sex matched healthy donors. Blood samples were collected from healthy donors and patients before surgery | In summary, we have confirmed that plasma IL-35 level and IL- 35 expression in the TILs of breast cancer tissues may be a vital factor in the development and prognosis of IDC. In the breast tumor microenvironment, IL-35+ Treg cells can induce Bregs while Breg derived IL-35 can also sustain Treg cells or CD8+ Tregs. Thus, in the IL-35-dependent regulatory milieu, these different IL-35- producing populations may promote reciprocal activity through a positive feedback mechanism, resulting in induction of iTr35 cells and IL-35+ Bregs through infectious tolerance. On the other hand, the increased induction of IL-35+ immunosuppressive populations in the TILs impairs the antitumor immune response (CD8 effector, CD4 effector, and B cells) and leads to breast cancer development and progression. | IL-35 | (67) |
| Adipose tissue inflammation in breast cancer survivors: effects of a 16-week combined aerobic and resistance exercise training intervention | pilot randomized study | breast cancer | F | no range (mean 53/55) | post | Non-Hispanic white, Hispanic | Participants were randomized to a supervised aerobic and resistance exercise intervention (EX Group) or delayed intervention control (CON Group). Endpoints were assessed at baseline and after the end of the 16-week intervention. Participants in the CON group were offered the exercise program following the study period. Participants in the EX group were supervised by a certified exercise specialist who adhered to and delivered the same prescribed exercise program, outlined in the Standard Operating Procedures trial manual, to ensure treatment fidelity. | A 16-week aerobic and resistance exercise intervention attenuates adipose tissue inflammation in obese postmenopausal breast cancer survivors. Future large randomized trials are warranted to investigate the impact of exercise-induced reductions in adipose tissue inflammation and breast cancer recurrence. | ATM M1, IL-6, TNF-alpha; ATM M2, adiponectin | (68) |
| Benefits in radical mastectomy protocol: a randomized trial evaluating the use of regional anesthesia | single-center, prospective, randomized clinical trial | breast cancer | F | 44-74 | NA | NA | Consenting patients were interviewed on the day of surgery, and the SF-36, DN4 and VAS scales were used. A blood sample was collected before to surgery. Anesthesia was performed according to group randomization (general anesthesia only or general anesthesia associated with SAM + PECS I), and the consumptions of fentanyl, propofol, cisatracurium and rocuronium were recorded. Patients were admitted to the post-anesthetic care unit (PACU) after surgery, and the following parameters were measured: time spent in PACU, VAS, PCA-morphine consumption, side effects and complications. Blood samples were collected 24 h after surgery, and VAS, PCA-morphine consumption, side effects and complications were assessed. Blood samples were evaluated in the ELISA assay to determinate the cytokine levels. | In conclusion, our results suggest that SAM + PECS I block in association with general anesthetic provides the most effective analgesia for radical mastectomy, but further studies are required. | IL-6, IL-10, IL-1 beta | (69) |
| Vitamin D Levels, Vitamin D Receptor Polymorphisms, and Inflammatory Cytokines in Aromatase Inhibitor-Induced Arthralgias: An Analysis of CCTG MA.27 | phase III adjuvant trial | breast cancer | F | 47.97-86.89 | NA | White | Within an 893-participant nested case-control AIA genome-wide association study, we nested a 72 AIA case-144 control assessment of vitamin D plasma concentrations, corrected for seasonal and geographic variation. We also examined 9 baseline inflammatory cytokines: interleukin (IL)-1β, IL-6, tumor necrosis factor-α, interferon (IFN)γ, IL-10, IL-12p70, IL-17, IL-23, and chemokine ligand (CCL)-20. Finally, we analyzed the multivariate effects of baseline factors: vitamin D level, previously identified musculoskeletal single nucleotide polymorphisms, age, body mass index, and vitamin D receptor (VDR) Fok-I variant genotype on AIA development. | In this nested case-control correlative study, vitamin D levels were not significantly associated with development of AIA; however, patients with the Fok-I VDR variant genotype were more likely to have a significant reduction in IL-1β level, and less likely to develop AIA. | examined 9 baseline inflammatory cytokines: interleukin (IL)-1β, IL-6, tumor necrosis factor-α, interferon (IFN)γ, IL-10, IL-12p70, IL-17, IL-23, and chemokine ligand (CCL)-20 | (70) |
| Prognostic impact of CD4-positive T cell subsets in early breast cancer: a study based on the FinHer trial patient population | randomized controlled trial | breast cancer | NA | no range | NA | NA | The study is based on the patient population of the randomized FinHer trial, where 1010 patients with early breast cancer were randomly allocated to adjuvant chemotherapy containing either docetaxel or vinorelbine, and human epidermal growth factor receptor 2 (HER2)-positive patients were also allocated to trastuzumab or no trastuzumab. Breast cancer CD4, FOXP3, and CXCL13 contents were evaluated using quantitative real-time polymerase chain reaction (qRT-PCR), and their influence on distant disease-free survival (DDFS) was examined using univariable and multivariable Cox regression and Kaplan-Meier estimates in the entire cohort and in selected molecular subgroups. Interactions between variables were analyzed using Cox regression. The triple-negative breast cancer (TNBC) subset of the HE10/97 randomized trial was used for confirmation | The results provide a high level of evidence that humoral immunity influences the survival outcomes of patients with early breast cancer, in particular of those with TNBC. | CXCL 13; FOXP3, CD4 | (71) |
| IL1 Receptor Antagonist Controls Transcriptional Signature of Inflammation in Patients with Metastatic Breast Cancer | clinical trial | breast cancer | F | NA | W | NA | Eleven female patients (Supplementary Table S5) with HER2− metastatic breast cancer received nab-paclitaxel (n = 3), eribulin (n = 5), or capecitabine (n = 2) along with anakinra 100 mg/day (FDA-approved dose for adults with rheumatoid arthritis; Kineret, Amgen Inc.) following 2-week treatment with anakinra only. | In summary, this study identifies an IL1-associated inflammatory signature in primary breast cancers that, if validated in follow-up clinical studies, could be used to stratify patients at diagnosis and justify use of ILl-directed therapies | IL-1beta | (72) |
| Reducing postsurgical exudate in breast cancer patients by using San Huang decoction to ameliorate inflammatory status: a prospective clinical trial | prospective clinical trial | breast cancer | F | no range (mean 52.8/51.87) | NA | NA | The study randomized 30 patients with breast cancer who fulfilled the inclusion and exclusion criteria to either a treatment (n = 15) or a control group (n = 15). Patients in the treatment group received liquid SHD, taken twice daily with or without food. Treatment was given for 1 day before surgery and for 7 days postoperatively. Participants in the control group received a placebo on the same schedule as the treatment group. | Perioperative treatment with SHD effectively lessened postoperative exudate and ameliorated inflammatory symptoms in patients who underwent surgery for breast cancer. | TNF-alpha, IL-6, IL-8, IL-2R | (73) |
| Atezolizumab Plus nab-Paclitaxel in the Treatment of Metastatic Triple-Negative Breast Cancer With 2-Year Survival Follow-up: A Phase 1b Clinical Trial | phase 1b clinical trial | breast cancer | F | 32-84 | NA | White, Black or African American, Asian, multiple, other | The 33 women had a median age of 55 years (range, 32-84 years) and received 1 or more doses of atezolizumab | In this phase 1b trial for metastatic triple-negative breast cancers, the combination of atezolizumab plus nab-paclitaxel had a manageable safety profile. Antitumor responses were observed, including in patients previously treated with a taxane | programmed heath-ligand 1, tumor-infiltrating lymphocytes, CD8; CD8+ T cell, CXCL 10 | (74) |
| The Anti-tumoral Effect of β-D-Mannuronic Acid (M2000) as a Novel NSAID on Treg Cells Frequency and MMP-2, MMP-9, CCL22 and TGFβ1 Gene Expression in Pre-surgical Breast Cancer Patients | phase II, randomized, controlled trial | breast cancer | F | NA | NA | Iranian | 24 women with BC were included in this study and were followed by fixed oral doses of M2000, 500 mg two times a day (6-8 weeks). Blood samples were collected at baseline and weeks 6-8. | Our findings demonstrated that M2000 therapy as a novel designed NSAID had valuable therapeutic effects on BC. No adverse effects were observed following the use of M2000 after 6-8 weeks. | MMP-2, MMP-9, CCL22, TGFbeta 1 | (75) |
| Sapylin (OK-432) alters inflammation and angiogenesis in vivo and vitro | prospective, consecutive cohort study | breast cancer | F | no range (46.03/47.9) | NA | NA | A prospective, consecutive cohort study included 120 patients diagnosed with breast cancer who underwent modified radical mastectomy was designed. Patients were randomized into two group, using or not using OK-432 (sixty patients per group) during surgeries. Patients’ drainage fluids were collected for three days after surgery. Inflammatory cytokines and chemokines were measured with ELISA assays. The proliferative, migratory, and angiogenic capacity of HUVEC and HFL1 cells HUVEC and HFL1 cells were measured after being treated with drainage fluids | Sapylin could stimulate the body to secrete a variety of cytokines to promote wound healing by promoting endothelial cell proliferation and migration, angiogenesis and by increasing fibroblast migration and collagen deposition. | IL-1a, IL-6, TGF-beta 1, VEGF/ HUVEC, HFL1 cells | (76) |
| A Large Randomized Trial: Effects of Mindfulness-Based Stress Reduction (MBSR) for Breast Cancer (BC) Survivors on Salivary Cortisol and IL-6 | randomized trial | breast cancer | F | No range (56.6/57.6) | NA | White non-Hispanic, Hispanic, other, more than one race/ethnicity reported | In the present study, BCS (N ¼ 322) were randomly assigned to a 6-week MBSR program for BC or usual-care control. Measurements of cortisol, IL-6, symptoms, and quality of life were obtained at orientation and 6 weeks. Cortisol and IL-6 were also measured prior to and after the MBSR(BC) class Weeks 1 and 6. | In summary, research has shown that MBSR is an effective intervention for reducing adverse psychological and physiological symptoms associated with cancer diagnosis or treatment among BCS (e.g., Henderson et. al., 2012; Hoffman et. al., 2012; Lengacher et. al., 2009; Lengacher et. al., 2016). The present study contributes to the biological evidence of the short-term effectiveness of MBSR(BC) on the objective markers of a stress hormone and an inflammatory cytokine. This body of research as a whole contributes to nursing research and practice by demonstrating that an alternative, non-pharmacological intervention can alleviate the stress response in the short term for BCS, providing more options for clinical interventions. | IL-6, salivary cortisol | (77) |
| The Influence of Single Nucleotide Polymorphisms and Adjuvant Radiotherapy on Systemic Inflammatory Proteins, Chemokines and Cytokines of Patients With Breast Cancer | clinical trial | breast cancer | F | 41-86 | NA | NA | Eighty-six female patients recovering from breast cancer surgery were investigated. As a control cohort, 82 healthy female blood donors were used. Blood-based SNPs, plasma C-reactive protein (CRP), cytokines and chemokines were analyzed for this purpose. | Dysregulation of immune responses, as indicated by plasma levels of CRP, CCL4 and IL2 were found in patients with breast cancer despite the removal of the tumour mass. The benefit of adjuvant RT, as indicated by reduced plasma amounts of inflammatory protein CRP and chemokine CCL5 were based on the SNPs of the patients. Analyses of blood-based SNPs, plasma CRP, IL2 and CCL5 are low cost, rapid and can be carried out using general laboratory facilities while requiring only a peripheral blood sample. The possibility of using these blood-based biomarkers as an indicator of patient immune status for selection of individual patient treatment warrants further investigation. | CRP, CCL4, IL-2; CRP, CCL5 | (78) |
| Transcriptomic profiles conducive to immune-mediated tumor rejection in human breast cancer skin metastases treated with Imiquimod | clinical trial | breast cancer | F | 44-71 | 62.5% are post-menopausal | NA | Ten patients were enrolled and treated with imiquimod for eight weeks | In conclusion, we have shown that topical imiquimod can induce a robust immune response in breast cancer metastases, and this response is more likely to occur in tumors with a pre-activated microenvironment. In this setting, imiquimod could be utilized in combination with other targeted immunotherapies to increase therapeutic efficacy. | Th-1 | (79) |
| T follicular regulatory cells suppress Tfh-mediated B cell help and synergistically increase IL-10-producing B cells in breast carcinoma | clinical trial | breast cancer | F | NA | NA | NA | All BC patients were newly diagnosed at the Second People’s Hospital of Dongying. Peripheral blood was obtained from all individuals once the written informed consent was received. All patients were untreated prior to sample collection. Controls were composed of 30 age matched females, who had no medical history of breast cancer or any other forms of malignancy. | Overall, this study demonstrates several distinctive features in circulating Tfr cells and suggests that Tfr cells may promote the formation of IL-10-producing B cells in BC. | Tfr-like, Treg-like, IL-10 | (80) |
| Low Plasma IL-8 Levels During Chemotherapy Are Predictive of Excellent Long-Term Survival in Metastatic Breast Cancer | prospective phase 2 trial | breast cancer | F | 32-75 | both | NA | We conducted an exploratory analysis of several circulating proteins, including IL-8, in the plasma. Plasma samples were obtained from 58 metastatic breast cancer patients who took part in a prospective phase 2 first-line bevacizumab chemotherapy trial. Samples were analyzed before therapy, after 6 weeks and 6 months of treatment, and at the final study visit. On the basis of a trajectory analysis of the plasma IL-8 levels, the patients were divided into 3 trajectory groups. | Low IL-8 levels during chemotherapy treatment might help identify patients with prolonged survival. | IL-8 | (81) |
| Cyclin E1 Expression and Palbociclib Efficacy in Previously Treated Hormone Receptor-Positive Metastatic Breast Cancer | clinical trial | breast cancer | F | NA | both | NA | The PALOMA-3 (ClinicalTrials.gov identifier: NCT01942135) trial randomly assigned 521 endocrine pretreated patients with metastatic breast cancer to receive palbociclib plus fulvestrant or placebo plus fulvestrant. Primary analysis was first conducted on 10 genes on the basis of pathway biology and evidence from previous studies followed by a systematic panel-wide search among 2,534 cancer-related genes. The association of gene expression with the effect of palbociclib on progression-free survival (PFS) was evaluated using Cox proportional hazards regression analysis, with gene expression as a continuous variable or dichotomized by median. | Addition of palbociclib to fulvestrant demonstrated efficacy in all biomarker groups, although high CCNE1 mRNA expression was associated with relative resistance to palbociclib. | CDK4, CDK6, cyclin D1, RB1 | (82) |
| Dose-dependent effect of aerobic exercise on inflammatory biomarkers in a randomized controlled trial of women at high risk of breast cancer | randomized controlled trial | Breast Cancer | F | no range (mean 34.4) | premenopausal | White/non-white | Participants were randomized to control (<75 min/wk; n=41), low-dose exercise (150 min/wk; n=38) or high-dose exercise groups (300 min/wk; n=37). The 5-menstrual-cycle-long home-based treadmill exercise intervention gradually increased minutes per week and intensity up to a maximum of 80% of age-predicted heart rate max | Moderate-to-vigorous aerobic exercise increased levels of pro-inflammatory biomarkers in a dose-dependent manner in a population of healthy women at high risk for developing breast cancer. These results suggest that for healthy premenopausal women, the mechanism of reduced breast cancer risk observed in physically active individuals may not be through reduced levels of inflammation. | CCL-2, IL-12, TNF-alpha; IL-10 | (83) |
| Phase I dose-escalation trial to repurpose propagermanium, an oral CCL2 inhibitor, in patients with breast cancer | multi-institutional, open-label, phase I study | breast cancer | F | 40-69 | NA | NA | Propagermanium was given from7 days before surgery to the final day except for the day of surgery. The PG dose was escalated in a “3 + 3 design”, with cohorts of 3 patients per dose level of 30, 60, and 90 mg/body/day followed by a cohort of 3 patients receiving 90 mg/body/day (Figure 1). Standard postoperative therapy was scheduled after PG treatment | Thus, PG was given safely and it is expected to have antimetastatic potential in BC. | CCL2, IL-6 | (84) |
| Autologous dendritic cells and activated cytotoxic T‑cells as combination therapy for breast cancer | phase I/II prospective study | breast cancer | F | NA | NA | NA | In the present study, DCs and activated lymphocytes (treated with IL‑12 and IL‑18) were isolated from the peripheral blood of patients with breast cancer, using a lysate of tumor tissue as antigen. The patients received the cells as part of adjuvant or neoadjuvant regimens (stage IV disease or progression). Evaluation of immunity was performed at 3 and 6 months after terminating immunotherapy. Evaluation of the disease‑free period was performed for 3 years after surgery. | Therefore, it may be concluded that it is reasonable to conduct anti‑suppressor therapy targeted against suppressor cell populations (41,42) or their mediators (e.g., targeted immunotherapy using monoclonal antibodies or immune cells) as the first stage and cellular immune‑stimulating antitumor therapy (cellular immunotherapy) as the second stage | CD19+ B cells, CD16+/CD56+ | (85) |
| Efficacy and safety of the therapeutic cancer vaccine tecemotide (L-BLP25) in early breast cancer: Results from a prospective, randomised, neoadjuvant phase II study (ABCSG 34) | prospective, randomized, multicenter, phase II trial | breast cancer | F | 25-83 | both | NA | A total of 400 patients with HER2-early BC were recruited into this prospective, multicenter, randomized 2-arm academic phase II trial. Patients received preoperative SoC treatment (chemotherapy or endocrine therapy) with or without tecemotide. Postmenopausal women with estrogen receptor (ER)þþþ, or ERþþ and Ki67 < 14%, and G1,2 tumours (‘luminal A’ tumours) received 6 months of letrozole. Postmenopausal patients with triple-negative, ER /þ/þþ and Ki67   14%, and with G3 tumours, as well as premenopausal patients, received four cycles of epirubicin/cyclophosphamide plus four cycles of docetaxel. Primary end-point was residual cancer burden (RCB; 0/I versus II/III) at surgery. Secondary end-points included pathological complete response (pCR), safety, and quality of life. | Tecemotide did not increase toxicity when compared to SoC therapy alone | MUC-1, RCB 0/I | (86) |
| Immunogenomic profiling and pathological response results from a clinical trial of docetaxel and carboplatin in triple-negative breast cancer | clinical trial | breast cancer | F | 42-61 | both | Caucasian, Black, other | Patients with clinical stages II/III TNBC received 6 cycles of docetaxel and carboplatin. The primary objective was to determine if neoadjuvant docetaxel and carboplatin would increase the pCR rate in TNBC compared to historical expectations. We performed whole-exome sequencing (WES) and immune profiling on pre-treatment tumor samples to identify alterations that may predict pCR. Thirteen matching on-treatment samples were also analyzed to assess changes in molecular profiles. | Neoadjuvant docetaxel and carboplatin resulted in a pCR of 45.7%. WES and immune profiling differentiated patients with and without pCR. | IDO-1, PD-L1, interferon gamma signaling, CTLA4, cytotoxicity, tumor inflammation signature, inflammatory chemokines, cytotoxic cells, lymphoid, PD-L2, exhausted CD8, Tregs, and immunoproteasome | (87) |
| Effects of an Exercise and Nutritional Intervention on Circulating Biomarkers and Metabolomic Profiling During Adjuvant Treatment for Localized Breast Cancer: Results From the PASAPAS Feasibility Randomized Controlled Trial | randomized controlled trial | breast cancer | F | No range (53.8/48.9) | both | NA | In the PASAPAS randomized controlled trial, 61 women beginning adjuvant chemotherapy for localized breast cancer were randomized in a 6-month program of weekly aerobic exercises associated with nutritional counseling versus usual care with nutritional counseling. In the present analysis of 58 women for whom blood samples were available, first, circulating levels of biomarkers (ie, insulin, insulin-like growth factor 1, estradiol, adiponectin, leptin, interleukin-6, and tumor necrosis factor α) were measured at baseline and 6-month follow-up. Changes in biomarkers were compared between exercisers (n = 40) and controls (n = 18) using mixed-effect models. Second, serum metabolites were studied using an untargeted 1H nuclear magnetic resonance spectroscopy, and orthogonal partial least squares analyses were performed to discriminate exercisers and controls at baseline and at 6 months. | The present analysis of the PASAPAS feasibility trial did not reveal any improvement in circulating biomarkers nor identified metabolic signatures in exercisers versus controls during adjuvant breast cancer treatment. Larger studies preferably in women with poor physical activity level to avoid ceiling effect, testing different doses and types of exercise on additional biological pathways, could allow to clarify the mechanisms mediating beneficial effects of physical exercise during cancer treatment. | insulin, insulin0like growth factor I, estradiol, adiponectin, leptin, IL-6, TNF alpha | (88) |
| Inflammation Mediates Exercise Effects on Fatigue in Patients with Breast Cancer | randomized controlled trial | breast cancer | F | no range (52.2/53.9/52.9) | both | Hi | Two hundred and forty women scheduled for chemotherapy were randomized to 16 wk of resistance and high-intensity interval training (RT-HIIT),moderate-intensity aerobic and high-intensity interval training (AT-HIIT), or usual care (UC). In the current mechanistic analyses, we included all participants with >60% attendance and a random selection of controls (RT-HIIT = 30, AT-HIIT = 27, UC = 29). Fatigue (Piper Fatigue Scale) and 92 markers (e.g., interleukin-6 [IL-6] and tumor necrosis factor α [TNF-α]) were assessed at baseline and postintervention. Mediation analyses were conducted to explore whether changes in inflammation markers mediated the effect of exercise on fatigue. | This study is the first showing that supervised RT-HIIT partially counteracted the increase in inflammation during chemotherapy, i.e., IL-6 and soluble CD8a, which resulted in lower fatigue levels postintervention. Exercise, including both resistance and high-intensity aerobic training, might be put forward as an effective treatment to reduce chemotherapy-induced inflammation and subsequent fatigue. | IL-6, CD8a | (89) |
| Effects of Ketogenic metabolic therapy on patients with breast cancer: A randomized controlled clinical trial | randomized controlled trial | breast cancer | F | no range (44.8/45.2) | NA | NA | A total of 80 patients undergoing treatment with chemotherapy were randomly assigned to KD or control group for 12 weeks | KMT in breast cancer patients might exert beneficial effects through decreasing TNF-a and insulin and increasing IL-10. KD may result in a better response through reductions in tumor size and downstaging in patients with locally advanced disease; however, more studies are needed to elucidate the potential beneficial effects of KD in patients with metastases | TNF-alpha, serum insulin | (90) |
| Biomarkers of response to camrelizumab combined with apatinib: an analysis from a phase II trial in advanced triple-negative breast cancer patients | open-label, randomized, parallel, non-comparative, two-arms, phase II trial | breast cancer | F | 32-64 | NA | NA | TILs, CD8+ T cells and PD-1/PD-L1 expression were evaluated in tumor samples by immunohistochemistry. 59 Cytokines/chemokines, growth factors, or checkpoint-related proteins, blood immune cell subpopulations were analyzed in blood samples by multiplexed bead immunoassays or flow cytometry. Correlation between biomarkers and clinical outcomes including ORR, progression-free survival (PFS), and overall survival (OS) was analyzed. | Higher baseline TILs or a greater increase of tumor-infiltrating CD8+ T cells during therapy, lower baseline plasma HGF/IL-8, a decrease of plasma IL-8, an increase of plasma TIM-3/CD152 during therapy, higher baseline CD4+ T cells or B cells proportion in blood are potential biomarkers for combinational anti-angiogenesis and immunotherapy in advanced TNBC patients | TILs, CD8+, TIM-3, CD152, CD4+; HGF/IL-8 | (91) |
| Phase II clinical trial using anti-CD3 × anti-HER2 bispecific antibody armed activated T cells (HER2 BATs) consolidation therapy for HER2 negative (0-2+) metastatic breast cancer | phase II clinical trial | breast cancer | F | 28-73 | NA | NA | HER2- metastatic breast cancer (MBC) patients received 3 weekly infusions of HER2 BATs and a boost after 12 weeks. | In heavily pretreated HER2-patients, immune consolidation with HER2 BATs after chemotherapy appears to increase the proportion of patients who were stable at 4 months and the median OS for both groups as well as increased adaptive and innate antitumor responses. Future studies combining HER2 BATs with checkpoint inhibitors or other immunomodulators may improve clinical outcomes. | interferon-γ immunospots, Th1 cytokines, Th2 cytokines, and chemokines | (92) |
| Effects of synbiotic supplementation on serum adiponectin and inflammation status of overweight and obese breast cancer survivors: a randomized, triple-blind, placebo-controlled trial | randomized, triple-blind, placebo-controlled trial | breast cancer | F | no range (56.56/58.31) | post | NA | Following a 2-week run-in period, 76 participants were randomly allocated into 2 groups of 38, to receive either synbiotic supplements or placebo. Patients in synbiotic or placebo groups received 1 capsule after lunch meal for 8 weeks. In addition, in order to control the energy and dietary intake, all the participants were given an LCD during the study for 8 weeks. | In conclusion, 8-week synbiotic consumption by overweight and obese postmenopausal BCSs had beneficial effects on adiponectin, TNF-α, and hs-CRP. | Adiponectin; TNF-alpha, hs-CRP | (93) |
| Resistance Exercise Modulates Kynurenine Pathway in Pancreatic Cancer Patients | randomized clinical trial | pancreatic cancer | not mentioned | no range (61.1/59.3/61.3) | NA | NA | In the SUPPORT study, adult pancreatic cancer patients were randomized to intervention programs of 6-month (1) a Supervised moderate-to-high-intensity progressive resistance training or (2) unsupervised Home-based resistance training, or (3) to a standard care patient Control group. | Supervised resistance exercise might positively regulate the Kynurenine pathway and downregulate the kynurenine/ tryptophan (indicative of IDO/TDO enzyme) levels, hence modulating the immune system | Serum kynurenine, kynurenine/tryptophan ratio, IL-6; tryptophan | (94) |
| The Impact of Preoperative Enteral Nutrition Enriched with Eicosapentaenoic Acid on Postoperative Hypercytokinemia after Pancreatoduodenectomy: The Results of a Double-Blinded Randomized Controlled Trial | double-blind randomized controlled trial | pancreatic cancer | M/F | no range (69/64) | NA | NA | Patients with resectable periampullary cancer were randomized into either the control group or the treatment group. Patients in the treatment group received oral supplementation (600 kcal/day) containing EPA for 7 days before surgery. Patients in the control group received isocaloric isonitrogenous standard nutrition (600 kcal/day) without EPA for 7 days before surgery. | The results of a double-blinded randomized controlled trial indicated that preoperative immuno-nutrition had no marked impact on the rates of postoperative hypercytokinemia or infectious complications after PD | Il-6, IL-1beta, TNF-alpha, CD4/8 | (95) |

Abbreviations include: NA (not applicable), wk (week/weeks), PDAC (pancreatic ductal adenocarcinoma) and ELISA (enzyme-linked immunosorbent assay).
